# SparCC co-occurrence networking reveals intracommunity dynamics of the microbiome following colorectal surgery

**DOI:** 10.1128/spectrum.03973-25

**Published:** 2026-02-24

**Authors:** Zachary A. Ziegert, Alexander Troester, Julia Frebault, Paolo Goffredo, Wolfgang B. Gaertner, Cyrus Jahansouz, Christopher Staley

**Affiliations:** 1 Division of Basic & Translation Research, Department of Surgery, University of Minnesota Medical School 12269, Minneapolis, Minnesota, USA; 2 Division of Colon and Rectal Surgery, Department of Surgery, University of Minnesota Medical School 12269, Minneapolis, Minnesota, USA; Cleveland Clinic Lerner Research Institute, Cleveland, Ohio, USA

**Keywords:** antibiotics, centrality, hub species, microbiome stability, topological parameters, wound healing

## Abstract

The intestinal microbiota plays a critical role in post-surgical wound healing following bowel resection; however, perioperative, prophylactic antibiotic administration may deleteriously affect it. We previously used 16S rRNA amplicon sequencing of stool samples to assess perioperative and longitudinal changes in the microbiome through 6 months in patients undergoing (i) colonoscopy after mechanical bowel prep (MBP) alone, (ii) non-resectional colorectal surgery after MBP with oral antibiotics and prophylactic intravenous antibiotics no longer than 24 h post-operative (surgical bowel prep [SBP]), and (iii) resectional colorectal surgery with SBP. Our objective in this study was to investigate the translational utility of SparCC co-occurrence networking to uncover biologically relevant patterns. Network topological parameters and hub species were calculated using NetCoMi, and permutational statistical tests were used to compare parameters. Network similarity among cohorts and time points generally matched changes in beta diversity, except in the resectional cohort, where all networks could not be differentiated statistically. Similarity in centrality measures among hub species was frequently significantly less similar than expected by chance and corresponded to an increased edge density and modularity, suggesting the latter parameters may reflect re-stabilization of the microbiome following surgery. We further noted the infrequently reported genera *Enterocloster* and *Ruthenibacter* were hub species during time points associated with surgical recovery, suggesting potentially novel roles for these genera in wound healing. *Streptococcus*, frequently implicated in surgical site infections at our center, was also frequently positively associated with *Blautia* throughout all networks, suggesting an increasing abundance of commensal bacteria serves as a prophylactic strategy.

**IMPORTANCE:**

This study employs the emerging approach of co-occurrence networking to assess ecological dynamics in the microbiome following colonoscopy and colorectal surgery. We expand upon applications of this approach to determine hub species and investigate clinically translational interpretations of network topological parameters in the context of recovery across three different trajectories of perturbation. Our results provide a context in which to interpret these network parameters biologically and represent a foundational step in beginning to quantitatively leverage network-based approaches to study microbial ecology. Furthermore, we identify network hub taxa that may play previously unexplored roles in wound healing.

## INTRODUCTION

The intestinal (gut) microbiota of humans is a diverse community of bacteria, archaea, fungi, viruses, and protists that interact amongst themselves and with the host to facilitate health and disease (1, 2). The microbiota plays a variety of critical roles, including protection from pathogens, training of the immune system, and digestion and fermentation of dietary products to provide essential vitamins and other metabolites (3, 4). The gut microbiota is also critically associated with wound healing, especially following bowel surgery (5). Microbially produced butyrate is a critical energy source for colonocytes, supporting viability and intestinal barrier integrity while reducing pro-inflammatory cytokines (6, 7). However, imbalances in the composition of the microbiota can lead to increased abundances of *Enterobacteriaceae*, *Enterococcus*, and *Pseudomonas*, which are collagenase producers that can impair wound healing, leading to post-surgical complications (8–10).

Nearly all surgical patients receive antibiotics in the perioperative period to prevent surgical site infections (SSIs) (11). However, this intervention negatively impacts the commensal microbiota, resulting in delays in return to a normal, luminal compositional distribution (12, 13). Mucosa-associated microbiota are also impacted, but rates of post-surgical complications, including anastomotic leak, do not appear to be greatly affected (14). Nevertheless, the rates of SSI have remained relatively unchanged over the last several decades, while there is increasing confidence that the gut microbiota plays a role in these complications (15–17). Thus, there is a critical need to evaluate the impact of perioperative interventions on the microbiota in both the acute surgical period, as well as longitudinally, to determine areas for intervention to improve wound healing and post-surgical outcomes.

For the last 5 years, our group has been assessing microbiome changes in the perioperative and follow-up periods in the Minnesota (MN) Microbiome in Colorectal Surgery Observational Study (MN MiCROS) (18, 19). To date, this single-center study has characterized baseline, perioperative, and longitudinal (up to 6 months) microbiomes of 81 patients undergoing colonoscopy, colorectal surgery without bowel resection (ventral rectopexy or trans-anal excision), or colorectal surgery with resection (colectomy or low anterior resection) to assess how varying degrees of iatrogenic and surgical treatments impact disturbance and recovery of the microbiota (20). We took the colonoscopy group (receiving mechanical bowel prep [MBP] alone) to represent minimal disturbance of the microbiota, bowel resection (receiving surgical bowel prep [SBP]) to represent a severe and persistent disruption to the microbiota, and surgery without bowel resection (receiving SBP) to reflect an intermediate course with moderate disturbance and somewhat faster recovery. Our objective was to utilize a co-occurrence networking approach to better interrogate intracommunity dynamics among the bacteria to assess the translational utility of this method, relative to more traditional microbiome analyses. Microbial co-occurrence networks have recently been used to study atopic dermatitis (21), inflammatory bowel diseases (22, 23), maternal obesity (24), and healthy individuals (25). However, results of these approaches have been complementary to traditional compositional analyses, only identifying particular taxa of interest that are correlated in a certain disease or modules of interacting taxa that may be of functional interest. Network topological parameters like closeness centrality may be used to identify hub or keystone species (26, 27), but the translational interpretations of other network parameters like clustering coefficient, modularity, and others have thus far been elusive. We hypothesize that network analysis and statistical evaluation of these parameters in the background of MN MiCROS will provide a promising new translational context to better define how network topology relates to microbial ecology and can be leveraged clinically.

## RESULTS

**Fig 1:**
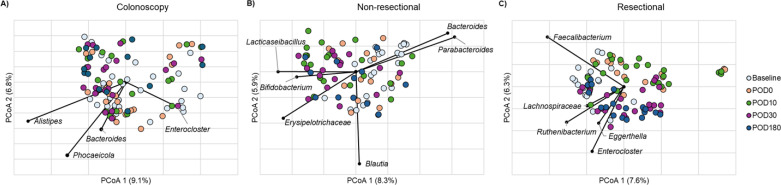
Principal coordinate analysis of Bray-Curtis dissimilarities among (**A**) the colonoscopy cohort, (**B**) the non-resectional cohort, and (**C**) the resectional cohort. Taxa identified as hubs in network analysis are overlaid.

Individual networks for each cohort and time point, including identification of hub species, are described in the Supplemental Material. Hub taxa identified among the colonoscopy group (Fig. 1A) did not correspond to changes in relative abundance, where *Ruminococcaceae*, *Blautia*, and *Streptococcus* showed significant, transient decreases at POD0, which recovered by POD10 (20). Instead, members of the Bacteroidales, including *Bacteroides*, *Phocaeicola*, and *Alistipes,* were hubs during post-procedural recovery (Table S1), with no strong hub taxa detected at baseline or POD180. Among the non-resectional group, *Bacteroides* and *Phocaeicola*, which decreased significantly at POD10, were identified as hubs at POD0, potentially suggesting a response to antibiotic ablation. Commensals, including *Bifidobacterium*, *Lacticaseibacillus*, and *Erysipelotrichaceae,* were identified as hub species at POD10 and POD30, suggesting potential roles for these in surgical recovery. Finally, among the resectional cohort, low-abundance members of the community, including *Ruthenibacterium* and *Eggerthella,* were hubs at POD0, while *Faecalibacterium* and *Lachnospiraceae* spp. were hubs during recovery at POD10 and POD30. *Enterocloster* was further identified as a hub at POD0 and POD30. Among surgical groups, expansion of *Enterococcus* and *Streptococcus* was observed following POD0 through POD30 (20), but neither of these became a hub, suggesting poor integration into the community.

### Within-group network comparisons mirror changes in beta diversity

Within the group of patients undergoing colonoscopy (i.e., MBP alone), a significant difference in beta diversity (Bray-Curtis dissimilarity) was observed between baseline and POD0 (analysis of similarity [ANOSIM] R = 0.28, *P* < 0.001; Fig. 1A). Similarly, community composition was significantly different between POD0 and POD30 (R = 0.18, *P* < 0.001), suggesting transient perturbation of the microbiome, with recovery of baseline composition in the first month. Among whole networks, baseline and POD0 networks had no statistically detectable similarity to each other (ARI = 0.007, *P* = 0.814), suggesting differences in topology, as supported by beta diversity analysis. However, the networks of all subsequent time points were statistically similar to baseline, and similarity increased with time (ARI = 0.074, 0.119, and 0.169, *P* = 0.023, 0.004, and <0.001 for POD10, POD30, and POD180), matching the recovery in alpha diversity previously observed (20). Network topological parameters did not differ significantly across all networks (Table 1); however, significant differences in centrality among central nodes were observed between the baseline network and either POD0 or POD10. Specifically, similarity in degree centrality was significantly less than expected by chance (*J* = 0.080 and 0.136, *P* = 0.003 and 0.035 relative to comparisons at POD0 and POD30), as were closeness (*J* = 0.118 and 0.120, *P* = 0.044 and 0.015) and eigenvector centrality (*J* = 0.038 and 0.091, *P* < 0.001 and = 0.009).

**TABLE 1:** Topological parameters for individual whole networks

Parameter	Group	Baseline	POD0	POD10	POD30	POD180
Number of components	Colonoscopy	3.00	8.00	2.00	3.00	1.00
	Non-resectional	2.00	1.00	2.00	3.00	1.00
	Resectional	2.00	1.00	4.00	1.00	3.00
Clustering coefficient	Colonoscopy	0.399	0.466	0.402	0.518	0.452
	Non-resectional	0.364	0.375	0.416	0.383	0.477
	Resectional	0.442	0.439	0.397	0.245	0.405
Modularity	Colonoscopy	0.226	0.227	0.201	0.111	0.106
	Non-resectional	0.223	0.201	0.322	0.245	0.144
	Resectional	0.236	0.233	0.301	0.336	0.170
Positive edge percentage	Colonoscopy	55.98	58.48	53.44	52.49	49.45
	Non-resectional	51.71	51.25	54.17	59.22	53.03
	Resectional	54.38	50.38	65.71	55.93	54.73
Edge density	Colonoscopy	0.171	0.140	0.214	0.246	0.296
	Non-resectional	0.167	0.228	0.137	0.168	0.309
	Resectional	0.177	0.212	0.143	0.096	0.198
Natural connectivity	Colonoscopy	0.0485	0.0415	0.0478	0.0632	0.0687
	Non-resectional	0.0421	0.0478	0.0350	0.0383	0.0690
	Resectional	0.0424	0.0449	0.0445	0.0288	0.0490

Among non-resectional patients (i.e., those receiving SBP), significant differences in beta diversity were observed between baseline and either POD10 or POD30 (R = 0.31 and 0.35, *P* < 0.001; Fig. 1B), suggesting durable disruption of the microbiome through the first month. Similar to the colonoscopy cohort, the baseline network and that at POD0 were considered random (ARI = 0.024, *P* = 0.469). In contrast to colonoscopy, networks remained random relative to baseline at POD10 and POD30 (ARI = −0.007 and 0.044, *P* = 0.872 and 0.119), supporting results from beta diversity analysis. Baseline and POD180 networks were statistically similar (ARI = 0.086, *P* = 0.013). Network modularity significantly increased at POD10 relative to baseline (Table 1, *P* = 0.023); however, no other statistically significant difference in topology was observed. Among central nodes, differences in betweenness centrality were significantly less than expected by chance at both POD10 and POD30 (*J* = 0.100 and 0.136, *P* = 0.018 and 0.035). Similarly, differences in closeness and eigenvector centrality were less than expected at POD10 relative to baseline (*J* = 0.154 and 0.048, *P* = 0.036 and 0.002).

In the resectional cohort (i.e., those receiving SBP with bowel resection), the baseline community differed from all subsequent time points (R = 0.36, 0.26, 0.24, and 0.24; *P* < 0.001; Fig. 1C). Furthermore, community composition at POD0 was significantly different than that at POD30 or POD180 (R = 0.30 and 0.34, *P* < 0.001), as was the community at POD10 (R = 0.26 and 0.24, *P* < 0.001), suggesting a prominent change in community composition after the first month. Unexpectedly, all networks were topologically statistically similar to baseline (ARI = 0.078, 0.066, 0.066, and 0.093, *P* = 0.016, 0.030, 0.013, and 0.013). Positive edge percentage was greater at POD10 than at baseline (Table 1, *P* = 0.007). At POD30, the clustering coefficient decreased while modularity increased (*P* = 0.007 and 0.015), and this time point reflected an inflection point in changes in beta diversity. Among central nodes, the difference in betweenness centrality at POD10 relative to baseline was less than expected (*J* = 0.045, *P* = 0.002).

### Between-group comparisons

**Fig 2:**
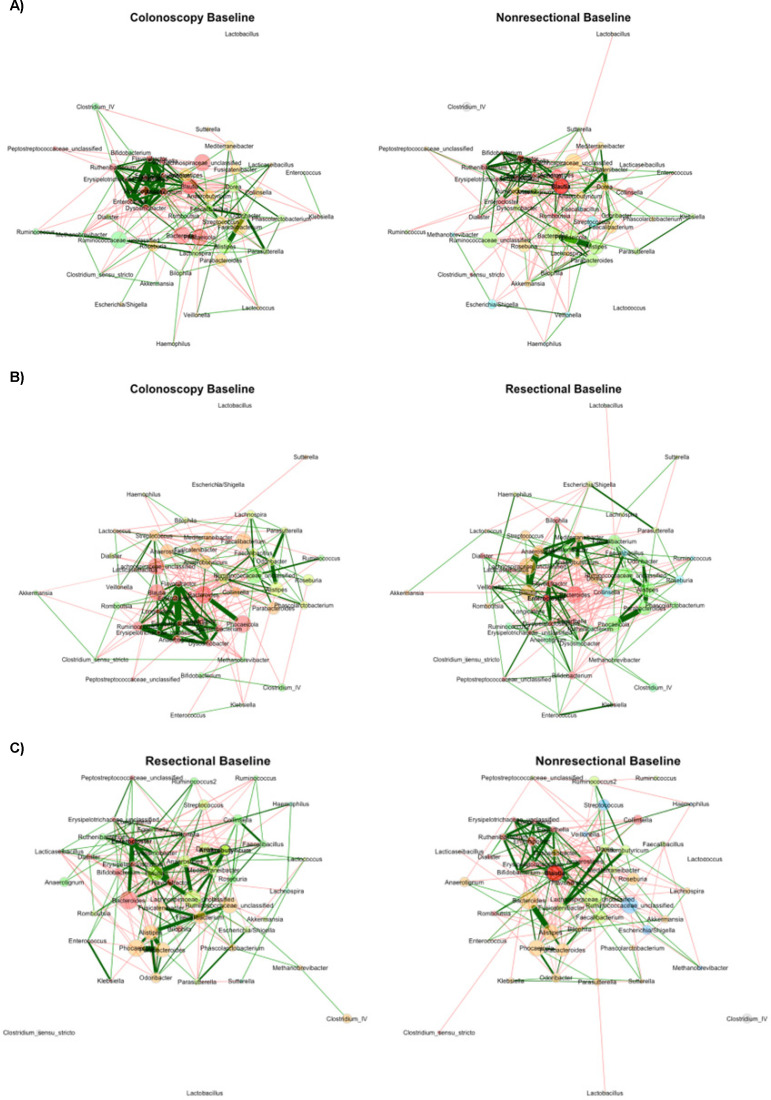
Comparisons of baseline networks. (**A**) Colonoscopy vs non-resectional networks; (**B**) colonoscopy vs resectional networks; (**C**) non-resectional vs resectional networks. Color indicates module assignment. Green lines reflect positive correlations and red lines reflect negative correlations; width reflects the strength of the relationship. Nodes are positioned in the same place in both networks being compared. Node sizes are based on centered-log ratio-transformed abundances.

At baseline, no differences in beta diversity were observed among all three groups (R = 0.014–0.089, *P* = 0.002–0.251) (20). The network from colonoscopy patients was statistically similar to those from non-resectional and resectional surgery groups (ARI = 0.064 and 0.065, *P* = 0.030 and 0.035), but those between non-resectional and resectional had no detectable statistical similarity (ARI = 0.050, *P* = 0.071; Fig. 2). Topological parameters did not vary significantly (Table 1). Among central nodes in each network, differences in betweenness centrality were less than expected between colonoscopy and resectional networks (*J* = 0.143, *P* = 0.046).

**Fig 3:**
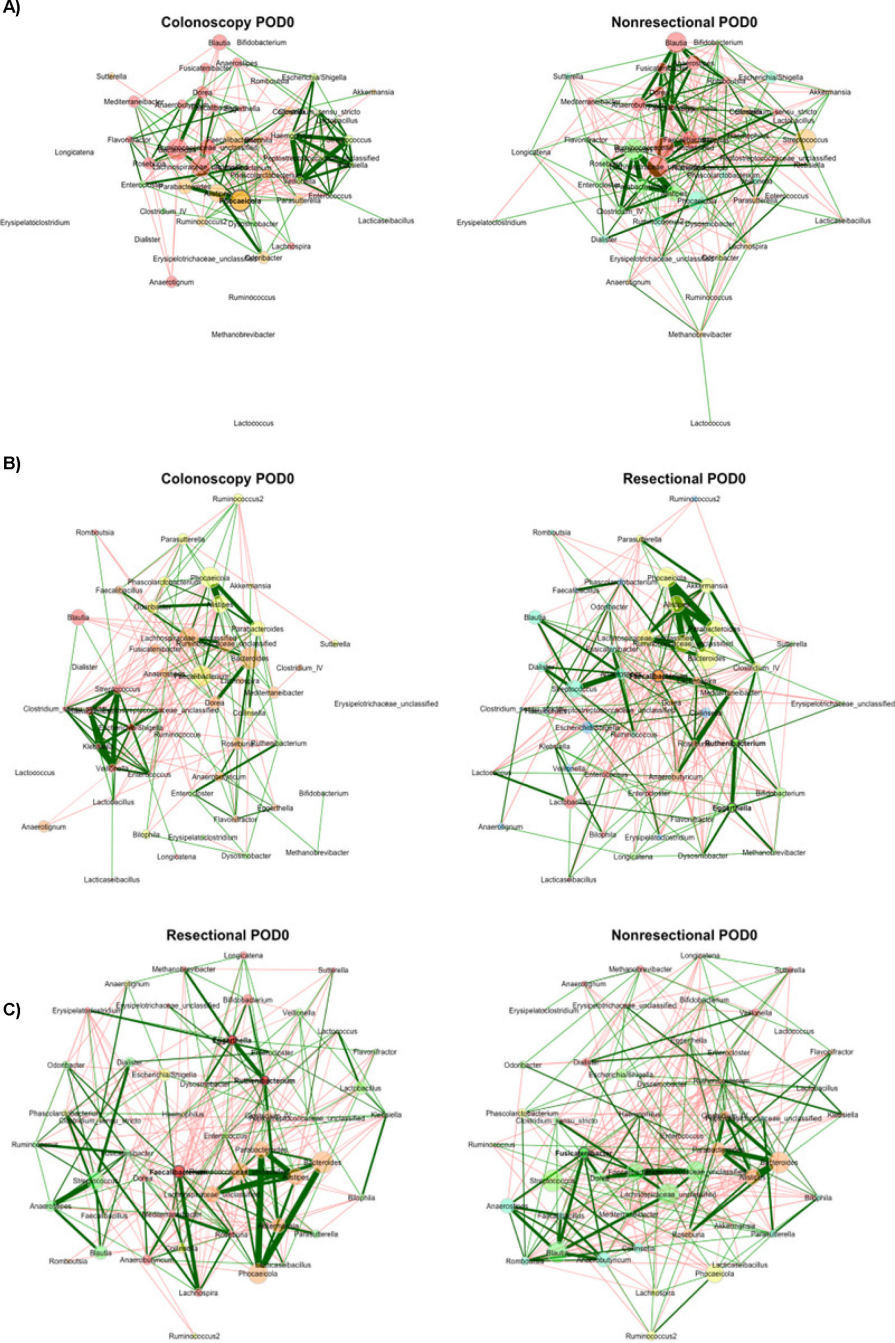
Comparisons of networks at POD0. (**A**) Colonoscopy vs non-resectional networks; (**B**) colonoscopy vs resectional networks; (**C**) non-resectional vs resectional networks. Color indicates module assignment. Green lines reflect positive correlations and red lines reflect negative correlations; width reflects the strength of the relationship. Nodes are positioned in the same place in both networks being compared. Node sizes are based on centered-log ratio-transformed abundances.

At POD0, both non-resectional and resectional community compositions were significantly different from the colonoscopy group (R = 0.365 and 0.384, *P* < 0.001), but did not differ significantly between surgical groups (R = 0.122, *P* = 0.005) (20). Colonoscopy networks had no statistically detectable similarity relative to non-resectional and resectional groups (ARI = 0.013 and 0.01, *P* = 0.650 and 0.780), similar to beta diversity analysis. Furthermore, networks from both surgery groups were random relative to each other (ARI = 0.015, *P* = 0.644; Fig. 3), while beta diversity differences did not meet the Bonferroni threshold. No significant differences in topological parameters were observed between colonoscopy and non-resectional networks; however, the resectional group had a significantly greater number of components (Table 1, *P* = 0.002) and lower positive edge percentage (*P* = 0.008), relative to colonoscopy. Among central nodes, differences in betweenness centrality between colonoscopy and resectional networks were less than expected (*J* = 0.143, *P* = 0.046).

**Fig 4:**
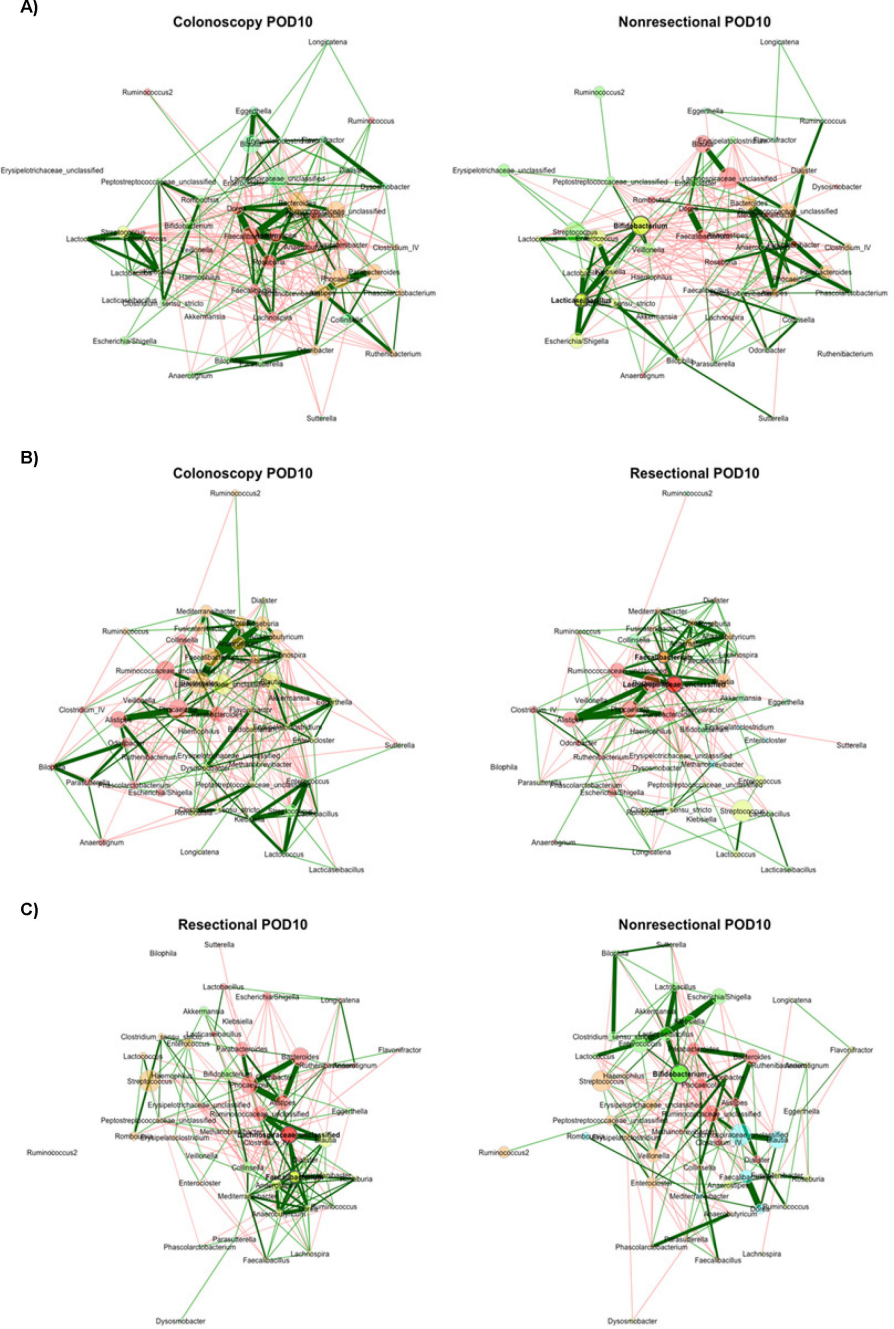
Comparisons of networks at POD10. (**A**) Colonoscopy vs non-resectional networks; (**B**) colonoscopy vs resectional networks; (**C**) non-resectional vs resectional networks. Color indicates module assignment. Green lines reflect positive correlations and red lines reflect negative correlations; width reflects the strength of the relationship. Nodes are positioned in the same place in both networks being compared. Node sizes are based on centered-log ratio-transformed abundances.

At POD10, beta diversity differed significantly between colonoscopy and the non-resectional group (R = 0.23, *P* < 0.001), but differences between colonoscopy and resectional surgery or between both surgical groups were not statistically significant after Bonferroni correction (R = 0.15 and 0.11, *P* = 0.006 and 0.002). The colonoscopy network was statistically similar to both non-resectional and resectional surgery groups (ARI = 0.104 and 0.102, *P* = 0.003 and 0.002) and also similar between the surgical groups (ARI = 0.081, *P* = 0.008; Fig. 4). In contrast to POD0, the resectional network had significantly greater positive edge percentage than colonoscopy (Table 1, *P* = 0.002). Among central nodes, the differences in both betweenness and eigenvector centralities were less than expected between colonoscopy and non-resectional networks (*J* = 0.083 and 0.048, *P* = 0.005 and 0.002). The difference in betweenness centrality was less than expected between colonoscopy and resectional networks (*J* = 0.091, *P* = 0.001), while the difference in eigenvector centrality was greater (*J* = 0.625, *P* = 0.016). Differences in degree, closeness, and eigenvector centralities were less than expected between surgical networks (*J* = 0.115, 0.130, and 0.045, *P* = 0.011, 0.026, and 0.002).

**Fig 5:**
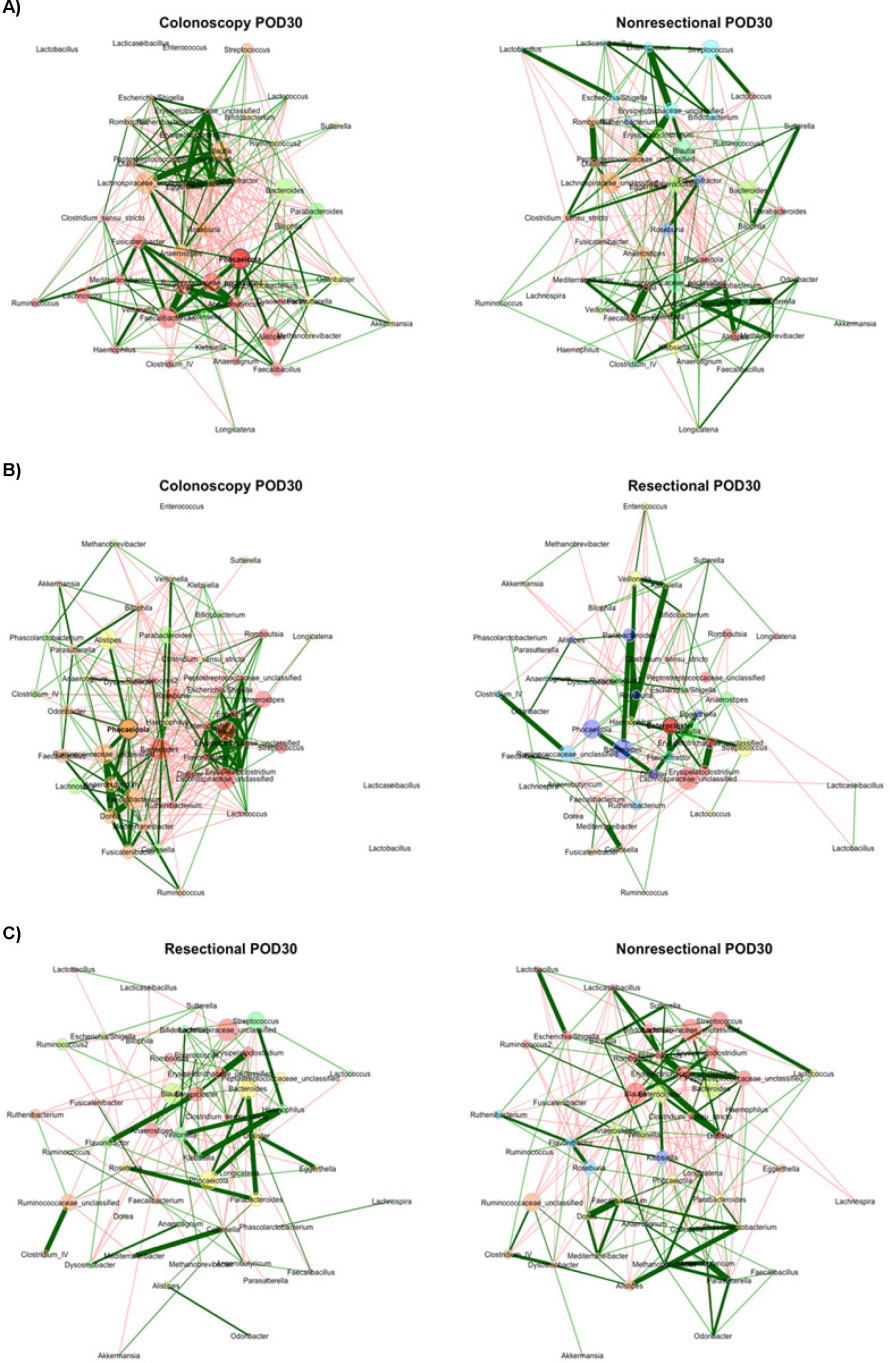
Comparisons of networks at POD30. (**A**) Colonoscopy vs non-resectional networks; (**B**) colonoscopy vs resectional networks; (**C**) non-resectional vs resectional networks. Color indicates module assignment. Green lines reflect positive correlations and red lines reflect negative correlations; width reflects the strength of the relationship. Nodes are positioned in the same place in both networks being compared. Node sizes are based on centered-log ratio-transformed abundances.

At POD30, community composition differed significantly between the colonoscopy group and both non-resectional and resectional surgery groups (R = 0.41 and 0.25, *P* < 0.001), but not between surgery groups (R = 0.05, *P* = 0.158). The colonoscopy network was statistically similar to both non-resectional and resectional networks (ARI = 0.095 and 0.102, *P* = 0.009 and 0.002), and networks from both surgery groups were topologically similar (ARI = 0.102, *P* = 0.002; Fig. 5). Similar to within-group analysis, the resectional network had greater modularity (Table 1, *P* = 0.003) relative to the colonoscopy network, but it also had a significantly lower clustering coefficient and edge density (*P* = 0.003 and 0.020). Among central nodes, differences in degree and closeness centralities were less than expected between colonoscopy and resectional networks (*J* = 0.130 and 0.071, *P* = 0.026 and 0.027).

**Fig 6:**
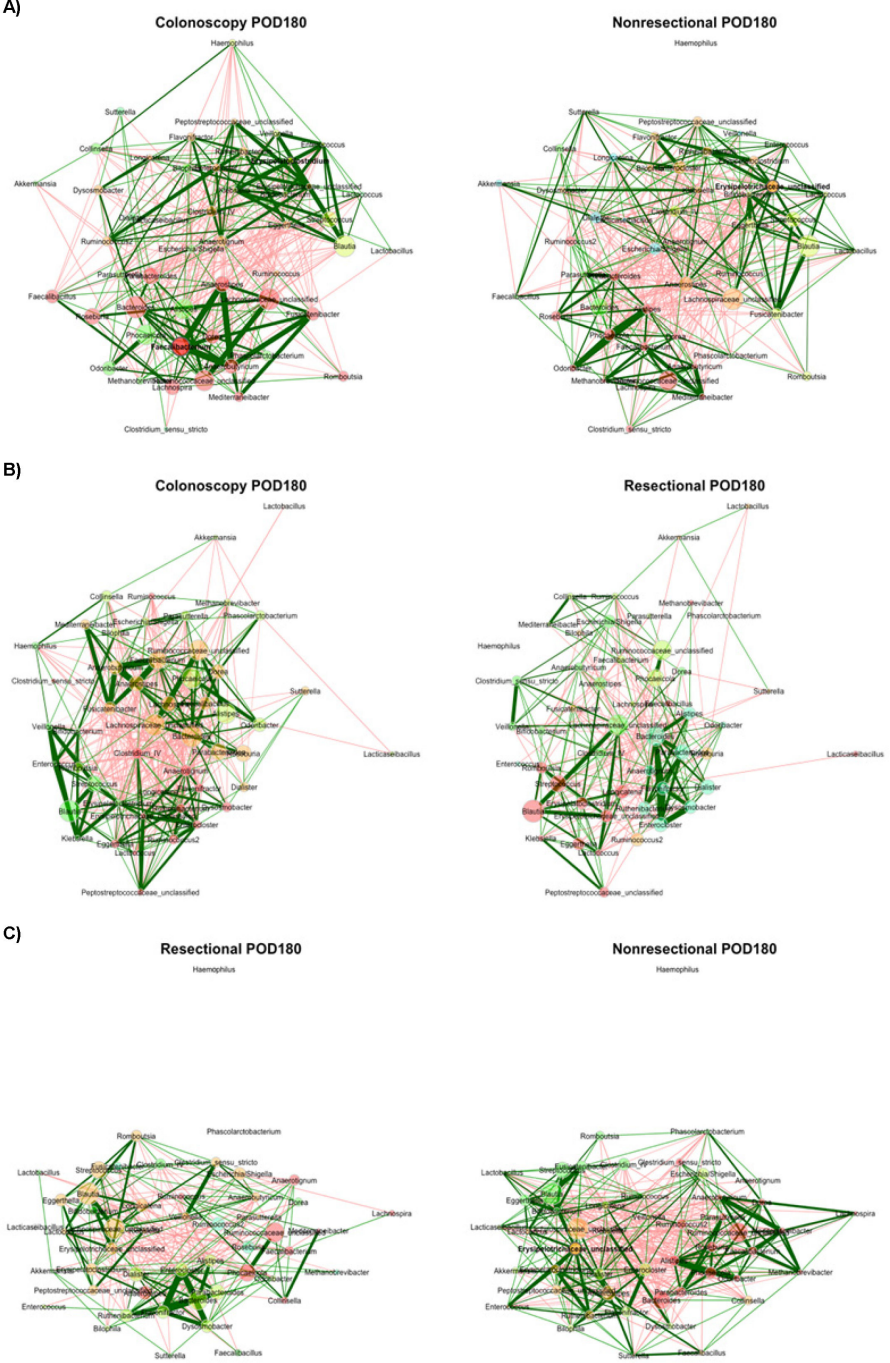
Comparisons of networks at POD180. (**A**) Colonoscopy vs non-resectional networks; (**B**) colonoscopy vs resectional networks; (**C**) non-resectional vs resectional networks. Color indicates module assignment. Green lines reflect positive correlations and red lines reflect negative correlations; width reflects the strength of the relationship. Nodes are positioned in the same place in both networks being compared. Node sizes are based on centered-log ratio-transformed abundances.

Finally, at POD180, community composition in the colonoscopy group did not differ significantly between that of non-resectional or resectional communities (R = 0.20 and 0.25, *P* = 0.007 and 0.001) or between surgery groups (R = 0.08, *P* = 0.110). However, while the colonoscopy and non-resectional networks were topologically similar (ARI = 0.099, *P* = 0.003; Fig. 6), the resectional network had no detectable statistical similarity relative to either of these (ARI = −0.001 and 0.015, *P* = 1.000 and 0.583). No significant differences in topological parameters were observed, but differences in betweenness centrality were less than expected between non-resectional and resectional nodes (*J* = 0.111, *P* = 0.033).

## DISCUSSION

The interpretation of networks and topological parameters as they relate to microbiome stability has recently been reviewed in depth (28). This study expands on this theoretical framework by incorporating statistical evaluation of network topological parameters, allowing more rigorous comparisons among groups and time points. For ease of interpretation, results have been summarized in Table 2.

**TABLE 2:** Summary of results relative to baseline samples^*a*^

Treatment	Measure	POD0	POD10	POD30	POD180
Colonoscopy	Beta diversity	**R = 0.28, *P* < 0.001**	R = 0.05, *P* = 0.159	**R = 0.18, *P* < 0.001**	R = 0.08, *P* = 0.151
	Network similarity	ARI = 0.007, *P* = 0.814; centrality less than expected	**ARI = 0.074, *P* = 0.023**	**ARI = 0.1149, *P* = 0.004**	**ARI = 0.169, *P* < 0.001**
Non-resectional	Beta diversity	R = 0.21, *P* = 0.001	**R = 0.31, *P* < 0.001**	**R = 0.35, *P* < 0.001**	R = 0.21, *P* = 0.015
	Network similarity	ARI = 0.024, *P* = 0.469	ARI = −0.007, *P* = 0.872; modularity increased; centrality less than expected	ARI = 0.044, *P* = 0.119; centrality less than expected	**ARI = 0.086, *P* = 0.013**
Resectional	Beta diversity	**R = 0.36, *P* < 0.001**	**R = 0.26, *P* < 0.001**	**R = 0.24, *P* < 0.001**	**R = 0.24, *P* < 0.001**
	Network similarity	**ARI = 0.078, *P* = 0.016**	**ARI = 0.066, *P* = 0.030**; positive edge percentage greater than baseline	**ARI = 0.066, *P* = 0.013**; clustering coefficient decreased; modularity increased	**ARI = 0.093, *P* = 0.013**

^*a*^ Samples shown in bold were statistically significant. Beta diversity results were evaluated against Bonferroni-corrected *α* = 0.00048.

In both colonoscopy and non-resectional surgery groups, network similarity generally matched differences in beta diversity, except for POD30 in the colonoscopy group, which may reflect stochastic variation in relative abundances of taxa that did not significantly impact community organization. We also observed that degree, closeness, and eigenvector centrality were also significantly less similar in these groups at time points following colonoscopy or surgery, respectively, and these time points (POD0 and POD10 in colonoscopy and POD10 and POD30 in non-resectional surgery) corresponded to a decrease, followed by an increase in edge density. This likely suggests a reorganization of connections within the community, although changes in edge density were not statistically significant. Higher edge density is suggested to increase community complexity (28), but the literature is divided as to whether this increases (29, 30) or decreases (31, 32) network stability. Furthermore, dissimilarity in closeness and eigenvector centrality may reflect changes in the relative importance of different taxa in the network (27), as was observed with changes in hub taxa. Given the context of recovery after surgery, we interpret these networks as becoming more stable as edge density increases, as supported by significantly lower edge density at POD30 in the resectional vs colonoscopy groups.

Surprisingly, all networks within the resectional group showed statistically significant similarity to each other, despite highly supported, statistically significant differences in beta diversity. Previous assessment of healthy human microbiome networks from different geographic regions indicated that human microbiome networks were significantly more similar and different from randomly generated networks (33). The authors suggested that the reason for this similarity was due to the necessity to maintain an organization that supported essential ecosystem processes (33), likely constrained by host–microbe interactions (34). In both surgical cohorts, modularity was found to increase significantly at POD10 (non-resectional) and POD30 (resectional) following the procedure. Modules in microbiome networks have been suggested to reflect niche partitioning (35), thus increased modularity may reflect changes in function associated with wound healing (36).

The interpretation of hub species, which may be intuitively thought to reflect critical members of the community, remains unclear without rigorous experimental validation (26). The majority of hubs identified reflect beneficial commensal microbiota (37); however, hubs identified do not correspond to the most abundant genera reported at each time point (20). Interestingly, *Eggerthella* and *Ruthenibacterium,* which are not commonly reported, were identified as hubs during resectional surgery. *Eggerthella*, along with *Collinsella*, is among the most abundant genera of Actinomycetota in the human microbiome (38), and *E. lenta* has been implicated in bacteremia (39). *Ruthenibacterium* is less well studied but is reported to be a lactate-producing genus isolated from stool (40). Similarly, *Enterocloster* was identified as a hub at several time points, including baseline and POD30, in the resectional cohort. Members of this genus inhabiting the gut have been shown to harbor inoviruses (41), which may foster immune tolerance (42) and reduce inflammation (43). Additional *in vitro* and *in vivo* work will be necessary to elucidate their specific role(s) in the community and during wound healing.

We were particularly focused on the associations of *Streptococcus* and *Enterococcus* in each network, as these are the genera predominantly implicated in SSI at our center (44). While *Streptococcus* was found to be a predominant member of several modules throughout the study, it was never identified as a hub species. Surprisingly, we also found it was relatively consistently and positively associated with *Blautia*. Abundances of *Blautia* were previously reported to predict greater concentrations of citrulline, which was suggested to be the mechanism underlying its protective effect against neutropenic fever in patients undergoing hematopoietic cell transplant (45). *B. producta,* along with *Clostridium boltae*, has further been shown to promote colonization resistance against vancomycin-resistant *Enterococcus* (46). There is no current literature to suggest cross-feeding between either *Streptococcus* or *Enterococcus*. The mechanistic dynamics of these associations will require further investigation. However, this finding suggests that optimal measures to protect against SSI should perhaps target increasing commensals resistant to infection rather than using antibiotics to ablate pathogens along with the commensal microbiota.

Studies using co-occurrence networking approaches to study microbial community interactions are inherently limited by a lack of standard workflows (27, 35). We chose to use NetCoMi (47) as a flexible and easy-to-use package implemented in R that allows for permutation-based statistical comparisons between networks and topological parameters. While previous studies compared networks of gut microbiome to random networks (33), they found that human gut microbiome networks were more similar to each other and less modular than random networks, leading us to directly compare gut microbiome networks to each other. There is also heterogeneity in the taxonomic composition of microbiomes within each group (20, 48), which may dampen the precision of the networks constructed (35). However, at least 25 samples per network have been suggested for robust construction (26, 27), and it is not feasible to collect this many samples from the same patient in a narrow temporal window, requiring us to pool demographically heterogeneous samples with differing exposures to diet, medication, etc. For our network construction, we chose to use all samples available, resulting in 25–30 samples per group. Inclusion of greater numbers of samples has been shown to improve precision (26), so we suspect it is unlikely to introduce bias.

In conclusion, we find that co-occurrence network analysis offers a complementary tool to expand the investigation of microbial intracommunity dynamics. We suggest that edge density may be a topological surrogate for community stability and that modularity increases during wound healing following surgery, suggesting functional diversification of the microbiome. We further identified *Ruthenibacterium* and *Enterocloster* as hub genera that may have previously unexpected and unexplored roles in wound healing and post-surgical recovery. Interestingly, we further noted positive associations with *Blautia* and potential pathogenic genera *Streptococcus* and *Enterococcus*, suggesting it may play a more direct role in limiting the expansion and pathogenic potential of these genera. These results must be interpreted cautiously as network properties are based on correlation, which may not accurately reflect underlying ecological dynamics (49). Thus, mechanistic elucidation of these dynamics will require dedicated *in vitro* and *in vivo* studies.

## MATERIALS AND METHODS

### Study design

Eighty-one adults were enrolled in an IRB-approved (University of Minnesota Study #00005429), prospective, observational cohort study we recently described (20) after providing written informed consent between March 2019 and April 2024. Patients underwent colonoscopy (*n* = 30), elective non-resectional (*n* = 25), or elective resectional surgery (*n* = 26) at the University of Minnesota Medical Center, and stool samples were collected at baseline (<30 days prior to procedure), during the procedure (POD0), and post-procedure within 10 days (POD10), 3–6 weeks (POD30), and at 6 months (POD180). Patient demographics and procedures are described elsewhere (20).

### 16S rRNA amplification, sequencing, and processing

Data for this project are available under BioProject accession number SRP250717, and details regarding DNA extraction, sequencing, and data processing were described previously (20). Briefly, DNA was extracted from fecal samples using the DNeasy PowerSoil Pro kit on the automated QIAcube platform using the inhibitor removal technology protocol (QIAGEN, Hilden, Germany). The V4 hypervariable region of the 16S rRNA gene was amplified and paired-end sequenced on the Illumina MiSeq platform (Illumina, Inc., San Diego, CA, United States) by the University of Minnesota Genomics Center (50). Sequence data were processed using mothur (ver. 1.41.1) (51, 52) and a modified version of our previously published pipeline (53). Processing included quality trimming, alignment against the SILVA database (54) (ver. 138_1) for operational taxonomic units (OTUs) clustering using 99% similarity and the OptiClust method (55), and taxonomic annotation against the Ribosomal Database Project (ver. 18) (56). Differences in community structure were evaluated using ANOSIM with Bray-Curtis dissimilarity matrices in mothur (57).

### Network construction and analysis

Microbiome data were filtered in mothur to remove taxa that were present at <10% of sequence reads and further cleaned in Excel to remove those with <20% prevalence across all samples. While there is no consistent prevalence threshold for network construction, 20% prevalence has been reported to effectively remove rare taxa while maintaining core microbial diversity (58, 59).

Co-occurrence networks, at the genus level, were constructed using NetCoMi (47) with SparCC (Sparse Correlations for Compositional data) (60). Networks were evaluated independently to assess topological parameters and hub taxa (classified to genus or the most resolved level), and then pairwise comparisons were made within groups (colonoscopy, non-resectional surgery, or resectional surgery) and between groups at each time point. Pairwise comparisons were done using the adjusted Rand index (ARI) (61) with the null hypothesis that ARI = 0 for two random networks. Topological parameters (e.g., centrality, modularity, Table 1) were also compared using the Jaccard index (*J*) (62, 63) among the most central nodes. ARI and Jaccard statistics were calculated in NetCoMi, and statistical comparisons were done using permutational tests at 1,000 iterations. Results were compared to ANOSIM (57) as a more traditional evaluation of community composition (beta diversity), calculated for all groups and time points together (Bonferroni corrected *α* = 0.00048). While statistical significance at this threshold was indicated by the software, *P* values were not calculated past three decimals to allow reporting of precise values.

Network construction was carried out in R (ver. 4.4.0) using the phyloseq (ver. 1.50.0), SpiecEasi (ver. 1.1.3), dplyr (ver. 1.1.4), ggplot2 (ver. 3.5.2), readxl (ver. 1.4.5), tibble (ver. 3.3.0), and NetCoMi (ver. 1.2.0) packages, along with other underlying dependencies. Mothur .shared and .constaxonomy files were passed as input to phyloseq along with metadata to create a phyloseq object. OTUs were assigned to genus, and networks were constructed using SparCC correlations (60) and centered-log ratio transformations using the netConstruct function in NetCoMi. Networks were clustered using fast greedy modularity optimization (64). Hub taxa were evaluated using degree, betweenness, and closeness centralities at a threshold of 0.9, as indicated in the software documentation. Source code is available in the Supplemental Material.

## Data Availability

Patient data were previously reported (20). Raw sequence data are available in the NCBI SRA under BioProject accession SRP250717. This research complies with the STORMS checklist (https://doi.org/10.5281/zenodo.18247827).

## SUPPLEMENTAL MATERIAL

Supplemental Material spectrum.03973-25-s0001.pdf Supplemental results, Figures S1 to S15, and Table S1.

Supplemental Text spectrum.03973-25-s0002.txt Source code for network analysis.

ASM does not own the copyrights to Supplemental Material that may be linked to, or accessed through, an article. The authors have granted ASM a non-exclusive, world-wide license to publish the Supplemental Material files. Please contact the corresponding author directly for reuse.
