# Supplementary material for: SparCC co-occurrence networking reveals intracommunity dynamics of the microbiome following colorectal surgery
Source: Microbiol Spectr. 2026 Feb 24;14(4):e03973-25. doi: 10.1128/spectrum.03973-25 (PMC13055225; doi:10.1128/spectrum.03973-25)
Supplement: Supplemental Material — Supplemental results, Figures S1 to S15, and Table S1. [file spectrum.03973-25-s0001.pdf]

Description of Individual Networks and Hub Taxa

Colonoscopy

Networks among the colonoscopy group underwent limited organizational changes throughout the study period. At baseline, three distinct modules were highly interconnected and predominantly comprised of members of the Bacteroidota and Bacillota (**Supplemental Figure S1**), with a fourth including *Akkermansia*, *Bifidobacteria*, and *Methanobrevibacter* that was poorly integrated into the network. No taxa were identified as hubs (**Table S1)**, likely due to strong positive associations within several modules. A reorganization of the network was observed at POD0 (**Supplementary Figure S2**), with more loosely defined modules containing multiple connected components. *Alistipes* became a hub within a Bacillota/Bacteroidota module, while another module reorganized to include *Lachnospiraceae* and *Bacteroides*, although these taxa were not designated as hubs. Interestingly, a third module formed that included *Blautia*, *Streptococcus*, and *Escherichia*, which were all positively associated. At POD10 (**Supplementary Figure S3**), five modules were distributed amongst two highly connected components, with *Bacteroides* designated as a hub for the network. By POD30 (**Supplementary Figure S4**), the network configuration began to resemble that at baseline, suggesting recovery of community structure, with *Phocaeicola* becoming a hub within a Bacillota/Bacteroidota module, while a second module formed around *Enterocloster*, including both *Streptococcus* and *Blautia*. A third, poorly connected module included *Parabacteroides*, *Bifidobacterium*, and *Methanobrevibacter*. At POD180 (**Supplementary Figure S5**), the *Phocaeicola*-dominated module remained, but no hubs were identified. *Escherichia* clustered in a peripheral module that also included *Akkermansia* and *Methanobrevibacter*, while the module that included *Blautia* showed strong, positive correlations with this genus and *Streptococcus*, as well as with *Klebsiella* and *Enterococcus*.

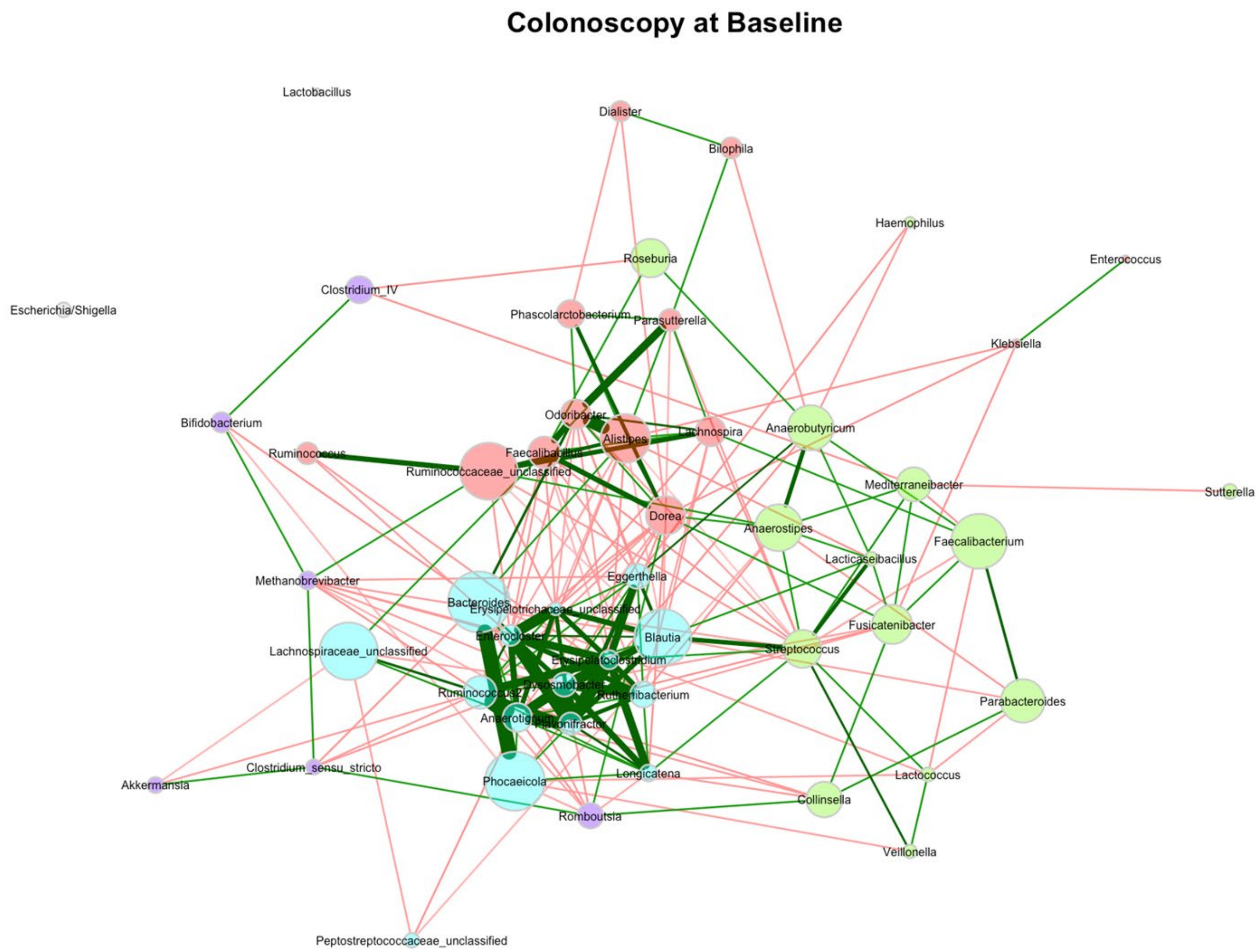

Supplementary Figure S1. SparCC network of baseline samples from patients undergoing colonoscopy. Node color reflects module assignment. Green lines reflect positive correlations and red reflect negative correlations; width reflects strength of the association. Node size indicates centered-log-ratio-transformed abundances.

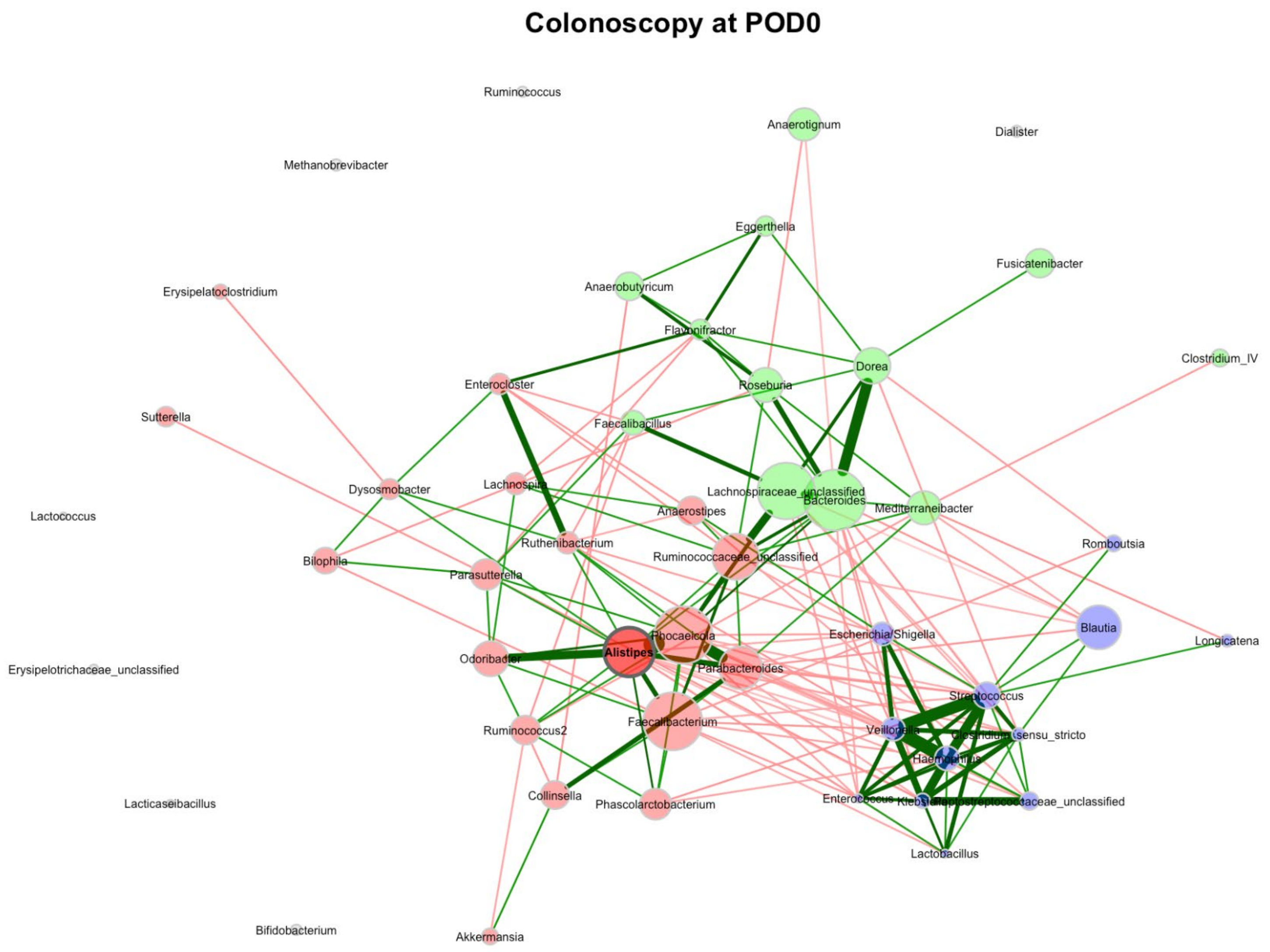

Supplementary Figure S2. SparCC network of POD0 samples from patients undergoing colonoscopy. Node color reflects module assignment. Green lines reflect positive correlations and red reflect negative correlations; width reflects strength of the association. Node size indicates centered-log-ratio-transformed abundances.

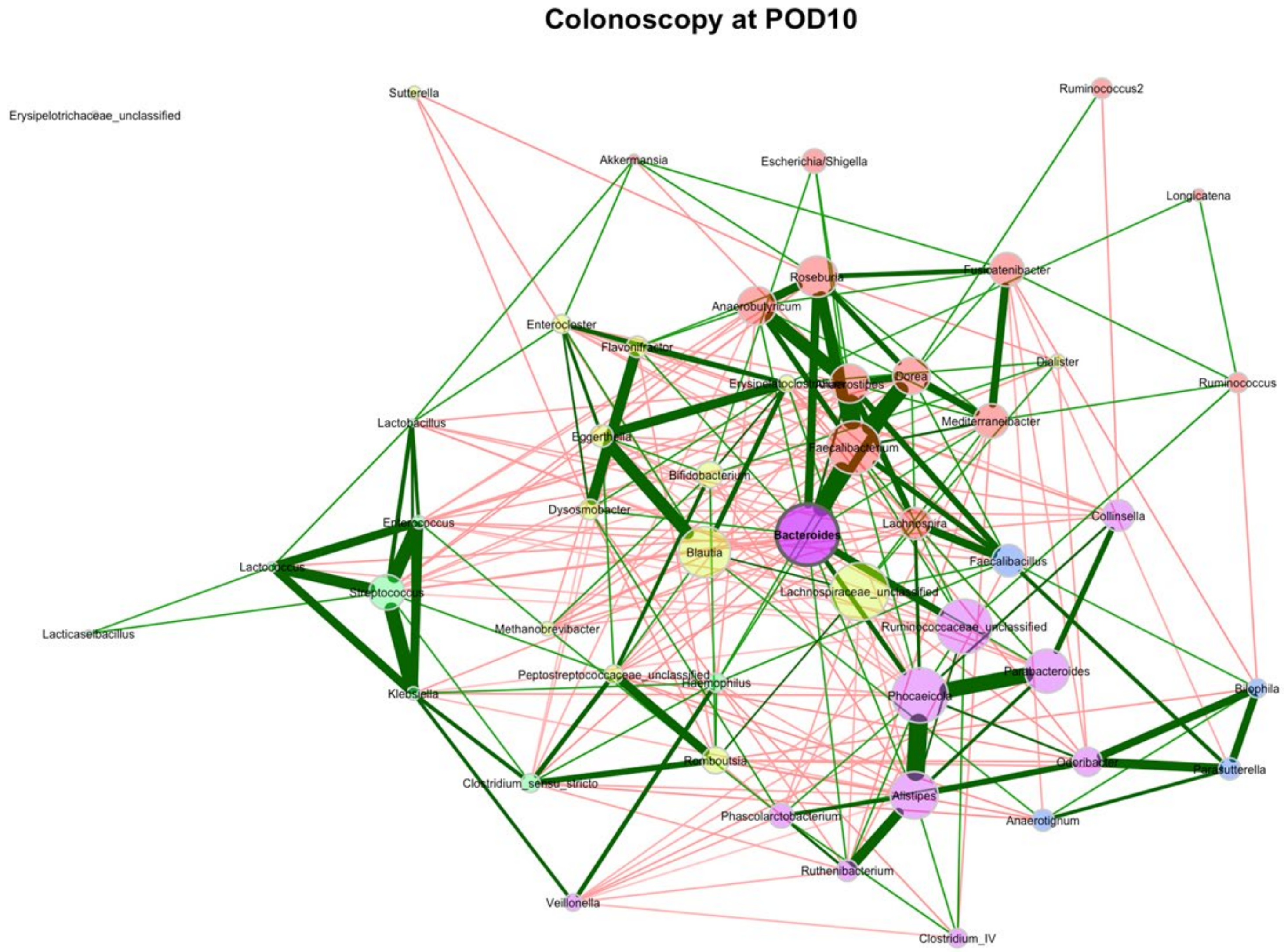

Supplementary Figure S3. SparCC network of POD10 samples from patients undergoing colonoscopy. Node color reflects module assignment. Green lines reflect positive correlations and red reflect negative correlations; width reflects strength of the association. Node size indicates centered-log-ratio-transformed abundances.

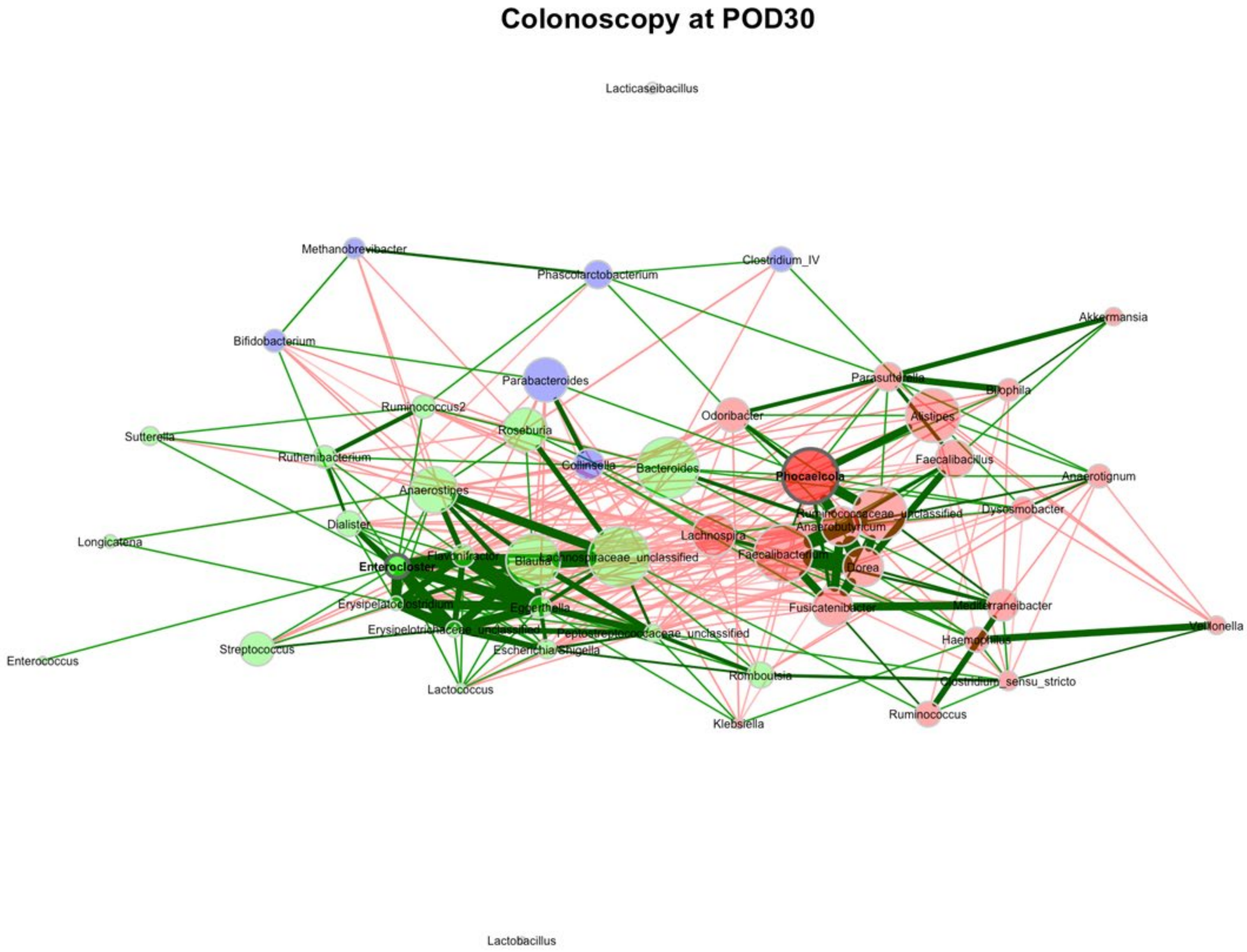

Supplementary Figure S4. SparCC network of POD30 samples from patients undergoing colonoscopy. Node color reflects module assignment. Green lines reflect positive correlations and red reflect negative correlations; width reflects strength of the association. Node size indicates centered-log-ratio-transformed abundances.

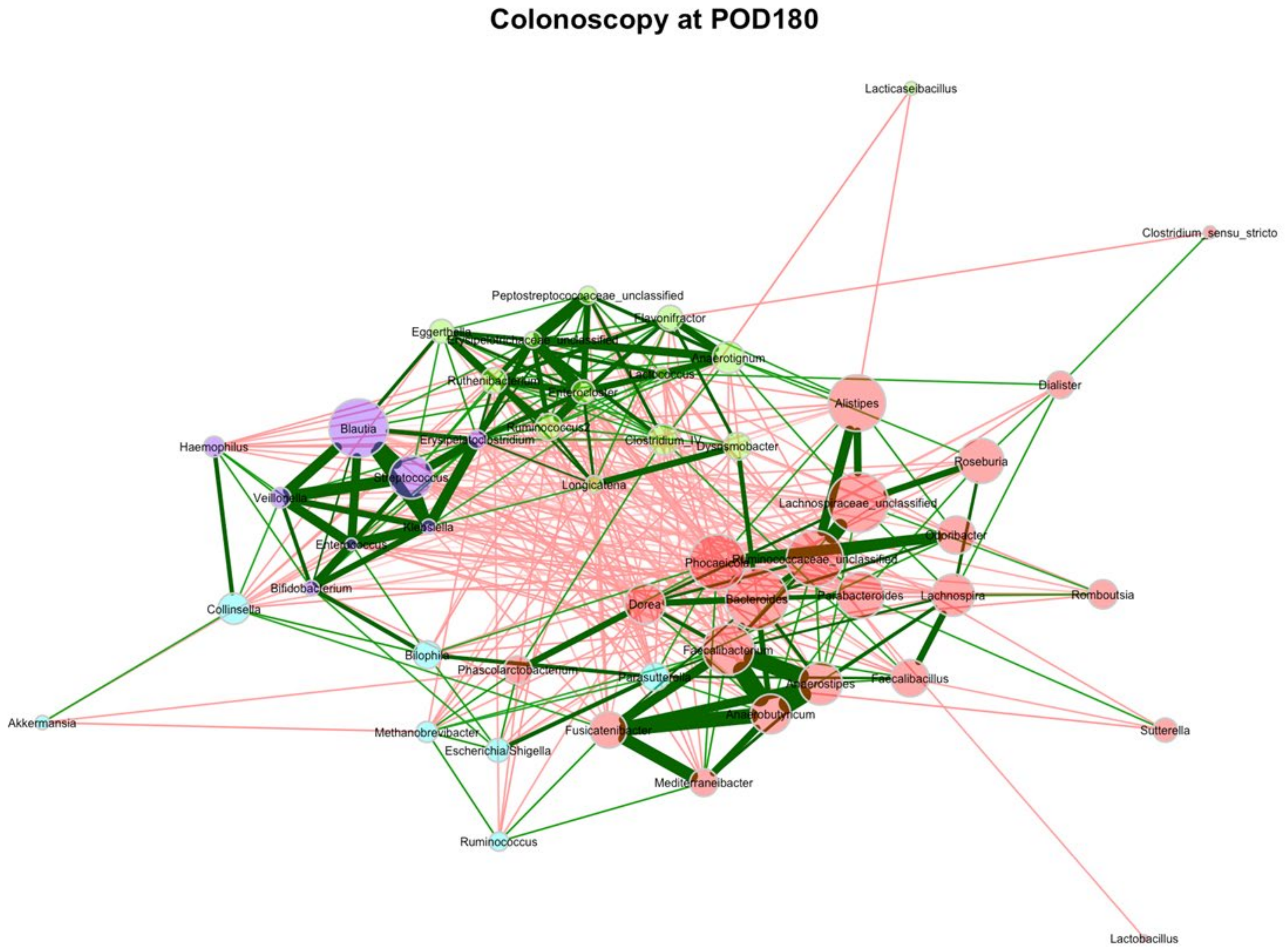

Supplementary Figure S5. SparCC network of POD180 samples from patients undergoing colonoscopy. Node color reflects module assignment. Green lines reflect positive correlations and red reflect negative correlations; width reflects strength of the association. Node size indicates centered-log-ratio-transformed abundances.

Among patients undergoing non-resectional surgery at baseline (**Supplementary Figure S6**), five modules were widely distributed across two highly connected components, with *Blautia* as a hub genus for a module consisting predominantly of Bacillota. Bacteroidota made up a second module, which included weak correlations with *Klebsiella. Streptococcus* and *Escherichia* were in a peripheral module, suggesting a minor role in community organization. At POD0 (**Supplementary Figure S7**), the Bacteroidota module became more prominent with both *Bacteroides* and *Parabacteroides* indicated as hubs. The Bacillota module was retained from baseline. *Escherichia* remained in a peripheral module while *Streptococcus* became the most abundant genus in a loosely connected module that also included *Phocaeicola*, *Akkermansia*, and *Methanobrevibacter*. At POD10 (**Supplementary Figure S8**), a prominent module that included *Bifidobacterium* and *Lacticaseibacillus* was highly connected, while Bacteroidota, Bacillota, and *Streptococcus* modules persisted. At POD30 (**Supplementary Figure S9**), the *Streptococcus* module expanded with further unclassified *Erysipelotrichaceae* as a hub for this module, which also included *Blautia*. Four other modules were loosely connected with the Bacteroidota module intact, while the Bacillota were more distributed throughout the network. At POD180 (**Supplementary Figure S10**), the network had resolved to four modules with Bacteroidota and Bacillota spread among two, and a third was predominantly comprised of just Bacillota. The fourth module included *Blautia* and *Streptococcus*, which were positively correlated.

Nonresectional at Baseline

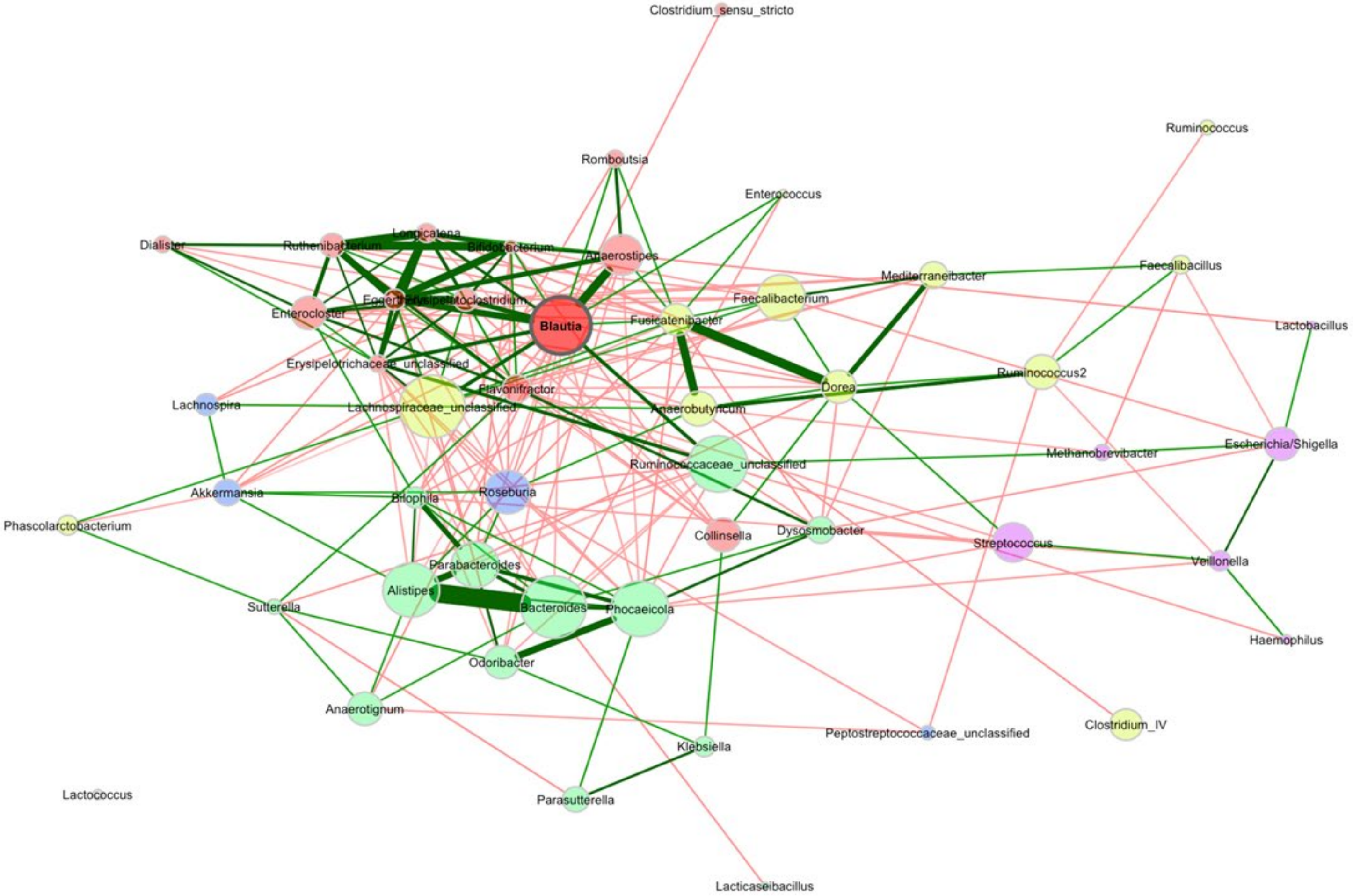

Supplementary Figure S6. SparCC network of baseline samples from patients undergoing non-resectional surgery. Node color reflects module assignment. Green lines reflect positive correlations and red reflect negative correlations; width reflects strength of the association. Node size indicates centered-log-ratio-transformed abundances.

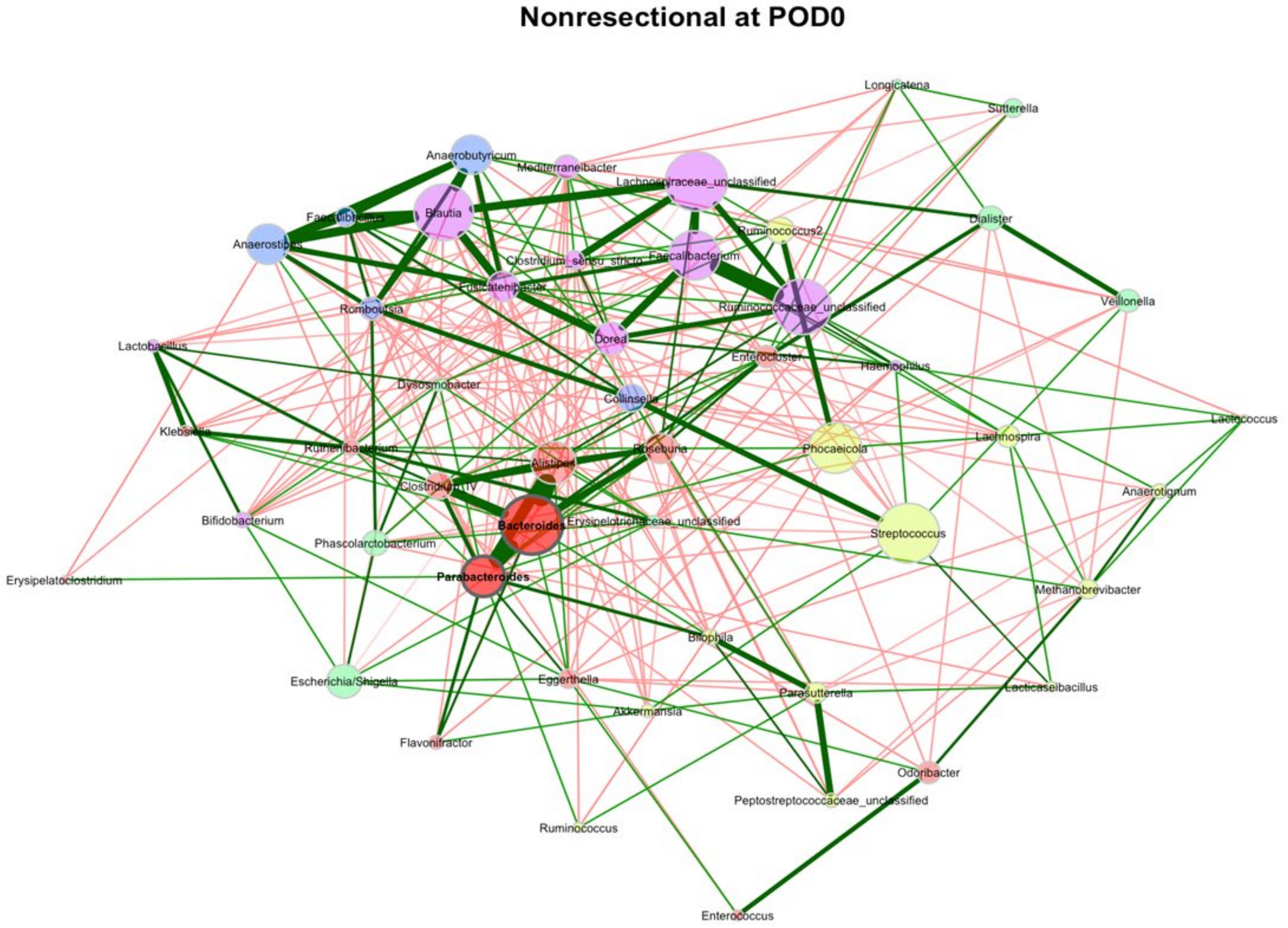

Supplementary Figure S7. SparCC network of POD0 samples from patients undergoing non-resectional surgery. Node color reflects module assignment. Green lines reflect positive correlations and red reflect negative correlations; width reflects strength of the association. Node size indicates centered-log-ratio-transformed abundances.

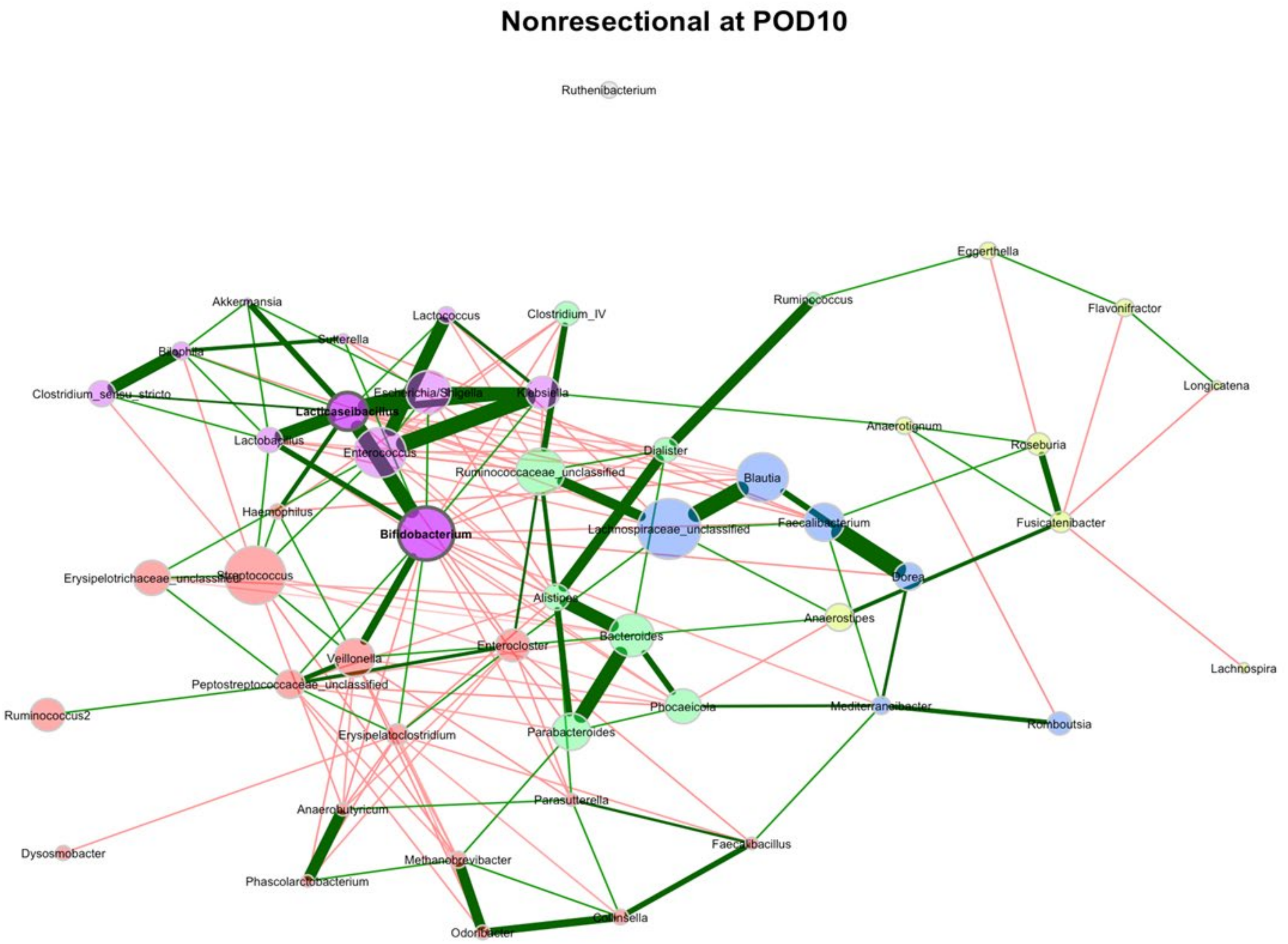

Supplementary Figure S8. SparCC network of POD10 samples from patients undergoing non-resectional surgery. Node color reflects module assignment. Green lines reflect positive correlations and red reflect negative correlations; width reflects strength of the association. Node size indicates centered-log-ratio-transformed abundances.

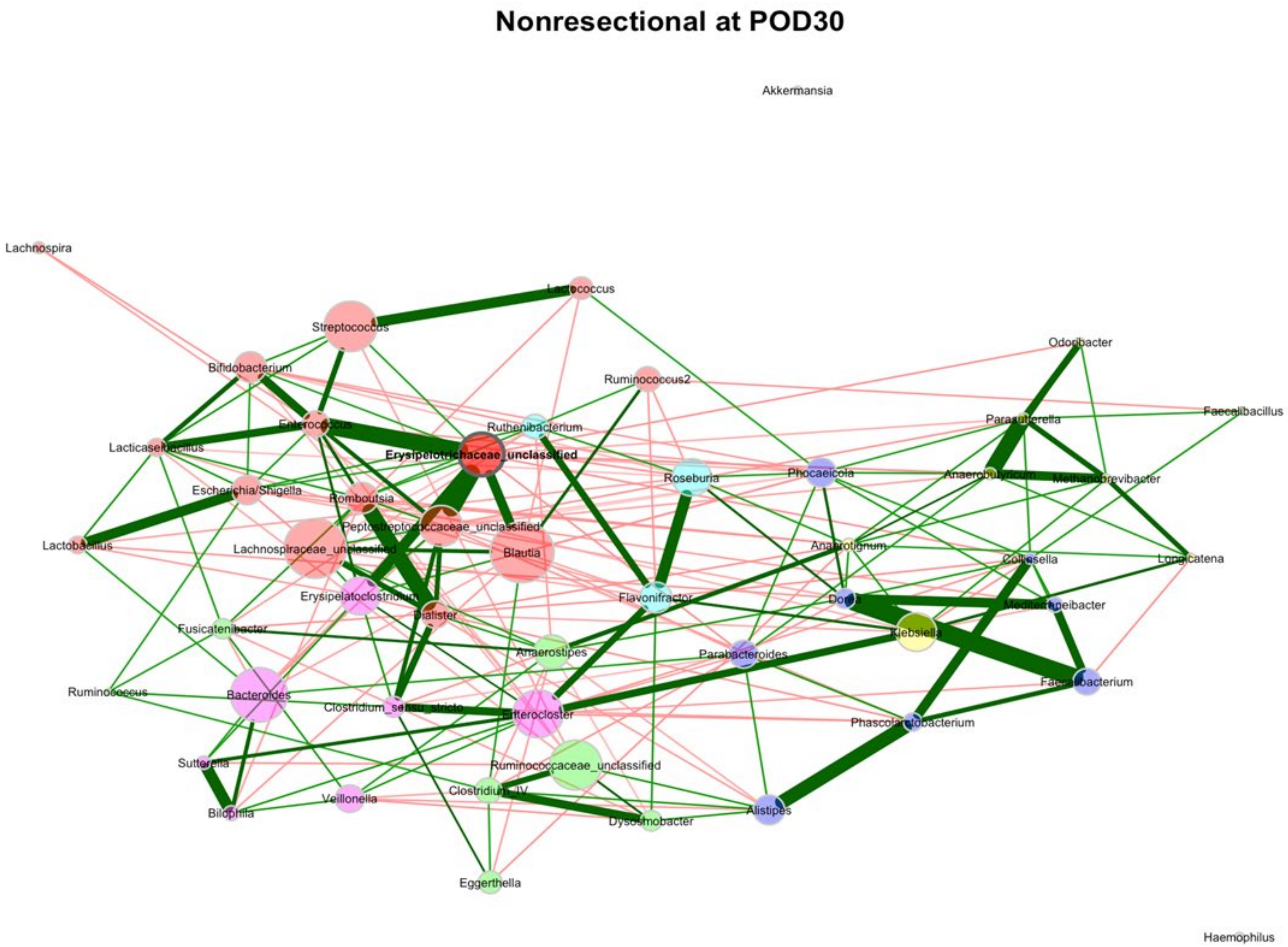

Supplementary Figure S9. SparCC network of POD30 samples from patients undergoing non-resectional surgery. Node color reflects module assignment. Green lines reflect positive correlations and red reflect negative correlations; width reflects strength of the association. Node size indicates centered-log-ratio-transformed abundances.

Nonresectional at POD180

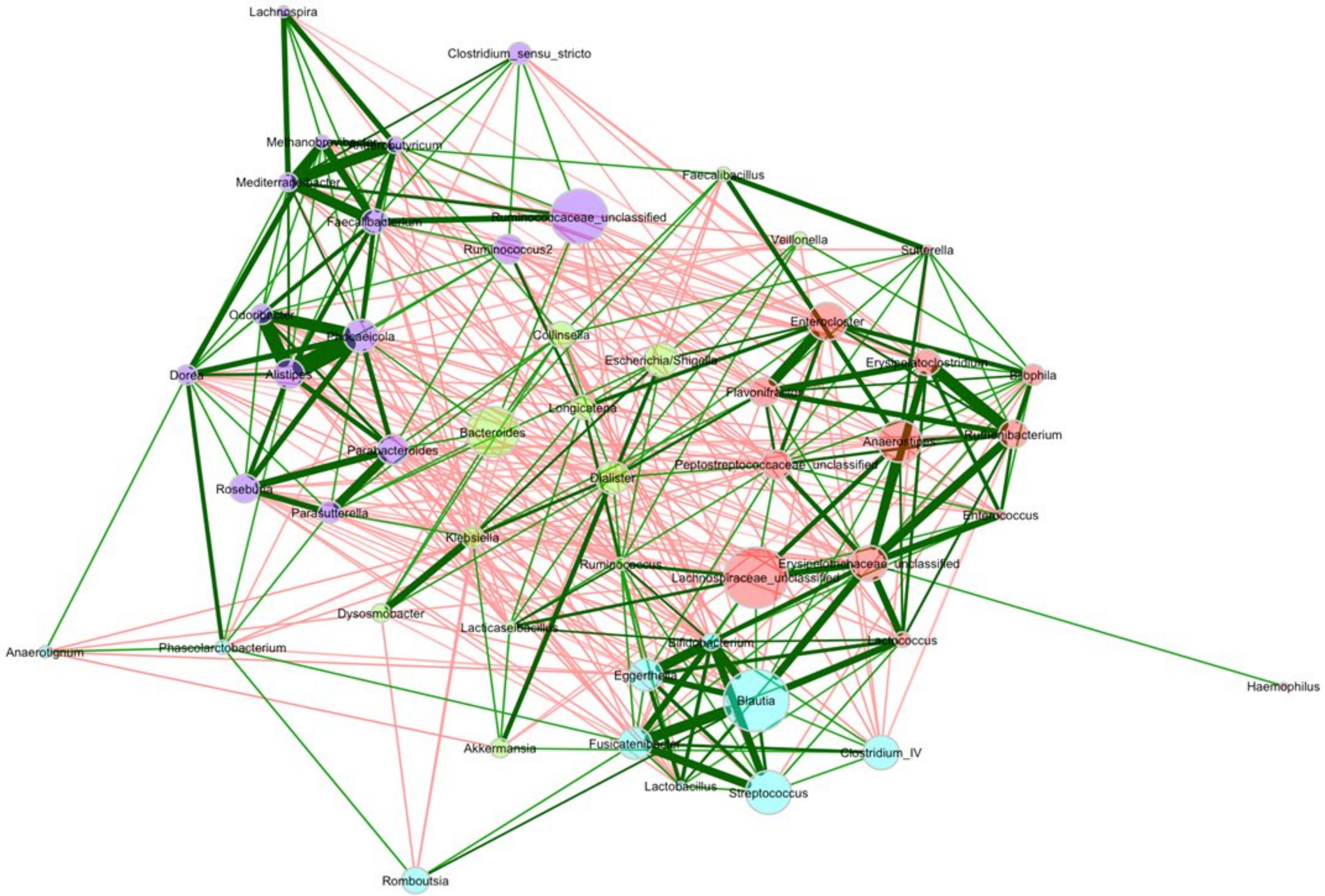

Supplementary Figure S10. SparCC network of POD180 samples from patients undergoing non-resectional surgery. Node color reflects module assignment. Green lines reflect positive correlations and red reflect negative correlations; width reflects strength of the association. Node size indicates centered-log-ratio-transformed abundances.

Among patients undergoing resectional surgery at baseline (**Supplementary Figure S11**), four modules comprised predominantly of Bacillota and Bacteroidota were loosely distributed amongst two highly connected components, with *Enterocloster* identified as a hub. *Enterococcus* and *Klebsiella* formed a fifth, peripheral module. At POD0 (**Supplementary Figure S12**), six weakly connected nodes were distributed across the network including a highly connected module that included *Bacteroides, Parabacteroides, Phocaeicola, Alistipes, Lachnospiraceae, Ruminococcaceae,* and *Akkermansia.* A prominent module also included *Streptococcus* and *Blautia*, which were positively associated, while *Eggerthella*, *Faecalibacterium*, and *Ruthenibacterium* were indicated as network hubs. At POD10 (**Supplementary Figure S13**), *Faecalibacterium* and *Lachnospiraceae* (not further unclassified) were network hubs and each was incorporated in its own, highly connected module. A third module was loosely connected and contained *Streptococcus*, *Enterococcus*, *Lactobacillus*, and *Bifidobacterium*. At POD30 (**Supplementary Figure S14**), *Enterocloster* became a network hub, with several small, but strongly connected modules. *Blautia* and *Streptococcus* remained positively associated and in the same module. At POD180 (**Supplementary Figure S15**), four modules were identified among three highly connected components. Two prominent modules were comprised of Bacteroidota and Bacillota, with one of these including *Blautia* and *Streptococcus*, which were strongly positively associated. However, no hub taxa were identified.

Resectional at Baseline

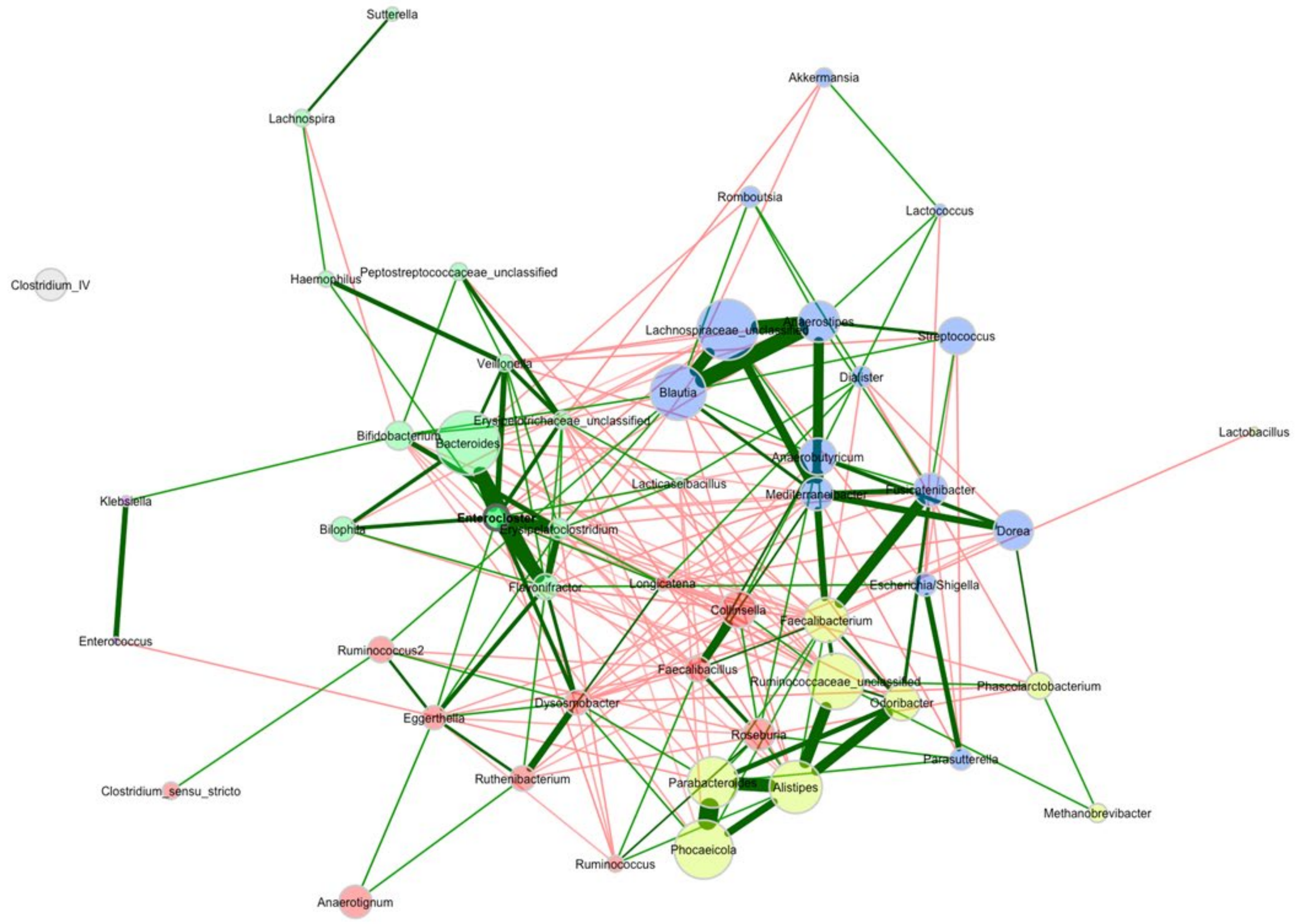

Supplementary Figure S11. SparCC network of baseline samples from patients undergoing resectional surgery. Node color reflects module assignment. Green lines reflect positive correlations and red reflect negative correlations; width reflects strength of the association. Node size indicates centered-log-ratio-transformed abundances.

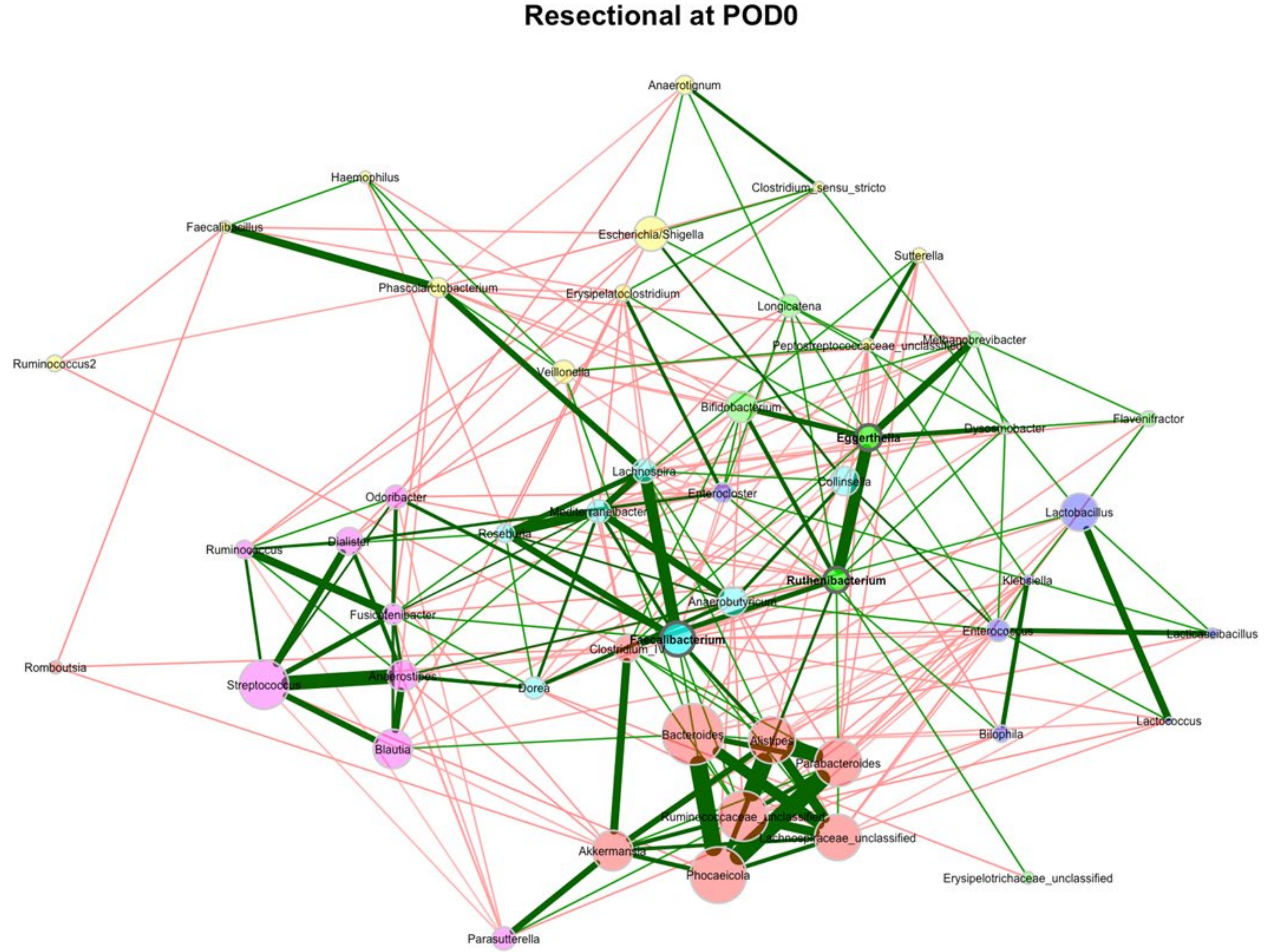

Supplementary Figure S12. SparCC network of POD0 samples from patients undergoing resectional surgery. Node color reflects module assignment. Green lines reflect positive correlations and red reflect negative correlations; width reflects strength of the association. Node size indicates centered-log-ratio-transformed abundances.

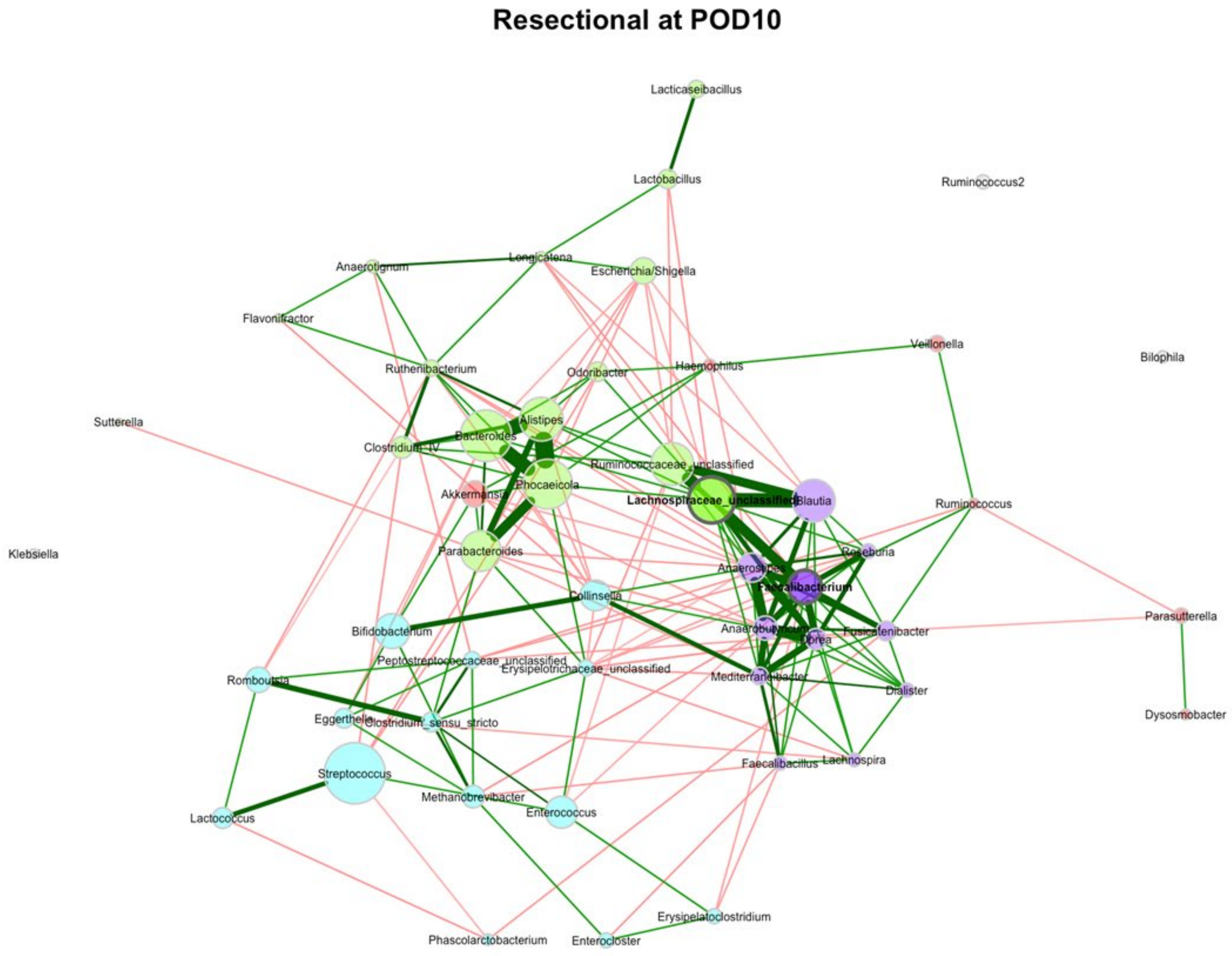

Supplementary Figure S13. SparCC network of POD10 samples from patients undergoing resectional surgery. Node color reflects module assignment. Green lines reflect positive correlations and red reflect negative correlations; width reflects strength of the association. Node size indicates centered-log-ratio-transformed abundances.

Resectional at POD30

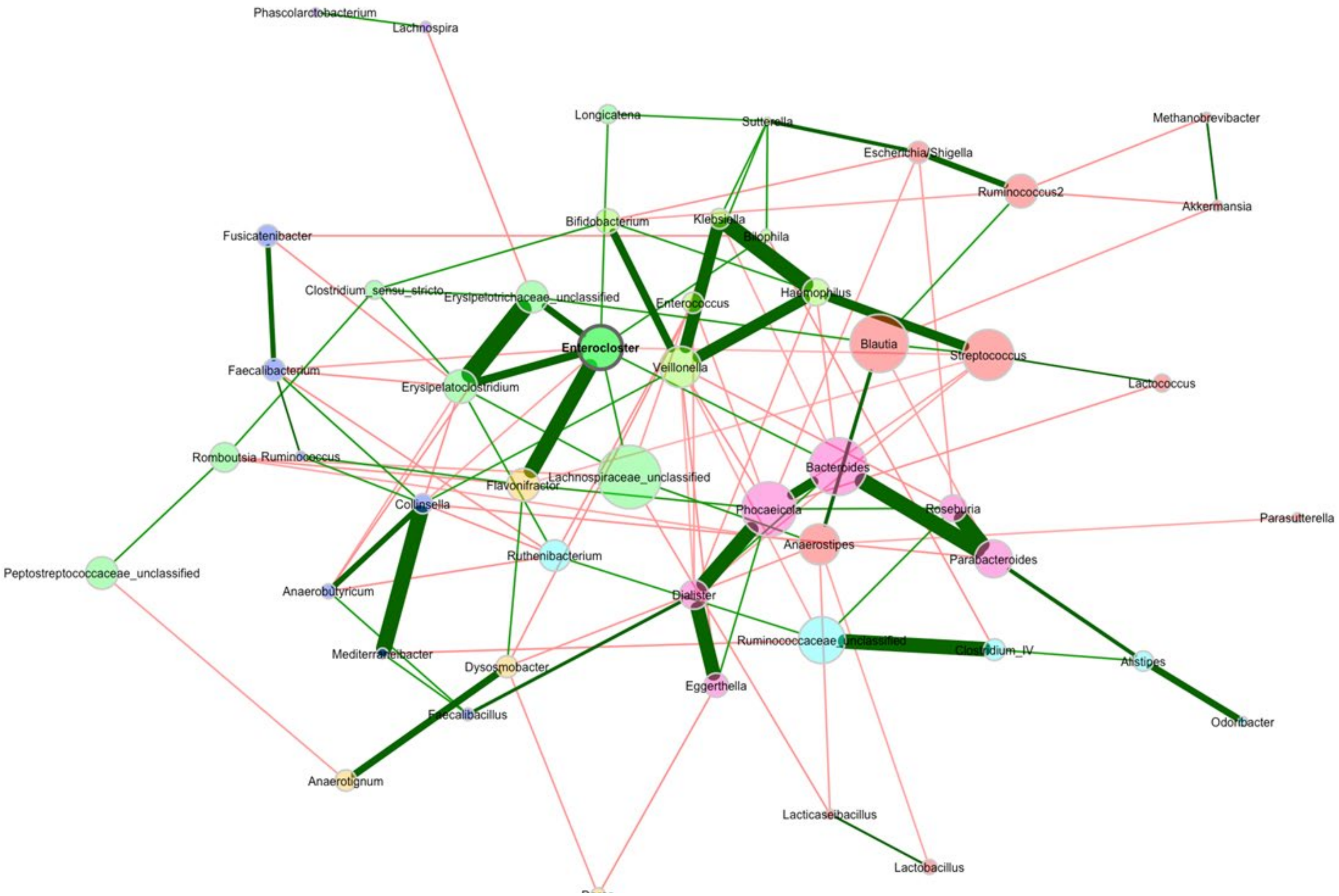

Supplementary Figure S14. SparCC network of POD30 samples from patients undergoing resectional surgery. Node color reflects module assignment. Green lines reflect positive correlations and red reflect negative correlations; width reflects strength of the association. Node size indicates centered-log-ratio-transformed abundances.

Resectional at POD180

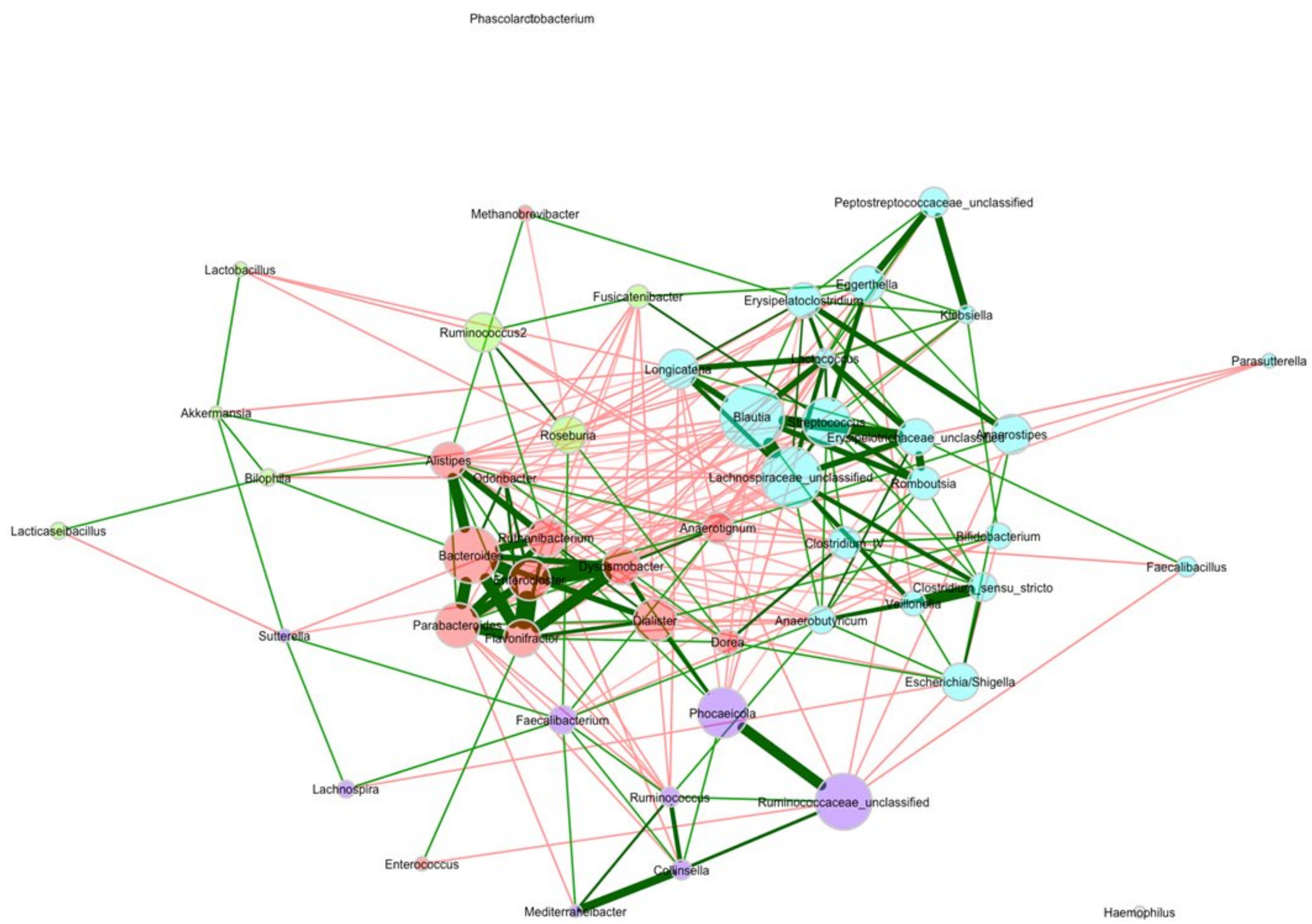

Supplementary Figure S15. SparCC network of POD180 samples from patients undergoing resectional surgery. Node color reflects module assignment. Green lines reflect positive correlations and red reflect negative correlations; width reflects strength of the association. Node size indicates centered-log-ratio-transformed abundances.

Table S1. Hub taxa in individual networks.

Group	Baseline	POD0	POD10	POD30	POD180
Colonoscopy	No hubs detected	*Alistipes*	*Bacteroides*	*Enterocloster*, *Phocaeicola*	No hubs detected
Non-resectional	*Blautia*	*Bacteroides*, *Parabacteroides*	*Bifidobacterium*, *Lacticaseibacillus*	*Erysipelotrichaceae* spp.	No hubs detected
Resectional	*Enterocloster*	*Eggerthella*, *Faecalibacterium*, *Ruthenibacterium*	*Faecalibacterium*, *Lachnospiraceae* spp.	*Enterocloster*	No hubs detected
